# Chemokines in diabetic eye disease

**DOI:** 10.1186/s13098-024-01297-w

**Published:** 2024-05-24

**Authors:** Xiongfeng Pan, Xinrui Tan, Judy McDonald, Atipatsa Chiwanda Kaminga, Yuyao Chen, Feizhao Dai, Jun Qiu, Kunyan Zhao, Yunlong Peng

**Affiliations:** 1 Pediatrics Research Institute of Hunan Province, Hunan Children’s Hospital, 86 Ziyuan Rd, Changsha, Hunan People’s Republic of China 410007; 2 The Affiliated Children’s Hospital of Xiangya School of Medicine, Central South University, Changsha, China; 3 Department of Pediatrics, The Second Xiangya Hospital, Central South University, Changsha, Hunan China; 4 McLaughlin Centre for Population Health Risk Assessment, University of Ottawa, Ottawa, ON Canada; 5 Department of Mathematics and Statistics, Mzuzu University, Mzuzu, Malawi; 6 Department of Ophthalmology, Guangdong Provincial People’s Hospital, Guangdong Academy of Medical Sciences, Guangzhou, China; 7 School of Public Health, University of South China, Hengyang, China; 8 Department of Epidemiology and Health Statistics, Medical College of Soochow University, Suzhou, China

**Keywords:** Chemokines, Diabetic eye disease, Inflammatory, Meta-analysis, Network meta-analysis

## Abstract

**Background:**

Diabetic eye disease is a common micro-vascular complication of diabetes and a leading cause of decreased vision and blindness in people of working age worldwide.Although previous studies have shown that chemokines system may be a player in pathogenesis of diabetic eye disease, it is unclear which chemokines play the most important role.To date, there is no meta-analysis which has investigated the role of chemokines in diabetic eye disease.We hope this study will contribute to a better understanding of both the signaling pathways of the chemokines in the pathophysiological process, and more reliable therapeutic targets for diabetic eye disease.

**Methods:**

Embase, PubMed, Web of Science and Cochrane Library systematically searched for relevant studies from inception to Sep 1, 2023. A random-effect model was used and standardized mean differences (SMDs) and 95% confidence intervals (CIs) were calculated to summarize the associated measure between chemokines concentrations and diabetic eye disease. Network meta-analysis to rank chemokines-effect values according to ranked probabilities.

**Results:**

A total of 33 different chemokines involving 11,465 subjects (6559 cases and 4906 controls) were included in the meta-analysis. Results of the meta-analysis showed that concentrations of CC and CXC chemokines in the diabetic eye disease patients were significantly higher than those in the controls. Moreover, network meta-analysis showed that the effect of CCL8, CCL2, CXCL8 and CXCL10 were ranked highest in terms of probabilities. Concentrations of CCL8, CCL2, CXCL8 and CXCL10 may be associated with diabetic eye disease, especially in diabetic retinopathy and diabetic macular edema.

**Conclusion:**

Our study suggests that CCL2 and CXCL8 may play key roles in pathogenesis of diabetic eye disease. Future research should explore putative mechanisms underlying these links, with the commitment to develop novel prophylactic and therapeutic for diabetic eye disease.

**Supplementary Information:**

The online version contains supplementary material available at 10.1186/s13098-024-01297-w.

## Introduction

Diabetic eye disease is a common micro-vascular complication of diabetes and a leading cause of decreased vision and blindness in people of working age worldwide [1]. Along with changes in lifestyle and increases in global prevalence of diabetes, the burden of diabetic eye disease has significantly increased over the past decade [2, 3]. An increasingly compelling body of work has emerged linking the disturbance of inflammatory reaction to the pathophysiological process of diabetic eye disease in the complex pathogenesis [4]. Specifically, patients with diabetic eye disease showed elevated inflammatory molecules which could have served as critical mediators of pathophysiological process of edema and neovascularization in the experiments [5]. Among these inflammatory molecules, recent studies indicated that chemotactic cytokines (chemokines) were critical molecules in modulating the immune microenvironment of diabetic eye disease [5]. Chemokines are important inflammatory, molecular families with four subfamilies (C, CC, CXC and CX3C; [6]. Remarkably, chemokines were critical players in the pathogenesis of mediating the secretion of inflammatory cytokines, coordinating immune cells transmigrate, and attracting immune cells to sites of ongoing inflammation of the vitreous and ocular fibrovascular membranes by following chemotactic gradients [5].

In addition to these pathways, another potential role of chemokines in diabetic eye disease has been supported in recent studies. Genetic work indicated that CC-chemokine gene polymorphism demonstrated an associated increase in susceptibility to diabetic retinopathy [7]. Furthermore, pre-clinical work indicated that chemokines were recognized as an initiator of inflammatory reactions, which increased in serum, vitreous and ocular fibrovascular membranes from patients with diabetic eye disease [8–10]. These chemokines may lead to retinal pathological changes by causing osmotic vascular damage [11, 12]. Thus, chemokines were potential candidates for orchestrating diabetic-eye, microenvironmental, inflammatory crosstalk, as well as linking the peripheral, vitreous and ocular fibrovascular membranes in the immune system.

Although previous studies have shown that chemokines system may be a player in pathogenesis of diabetic eye disease, it is unclear which chemokines play the most important role. To date, there is no meta-analysis which has investigated the role of chemokines in diabetic eye disease. The specific challenge is that the nomenclature of chemokines is not uniform, and there are currently about 50 chemokines acting on 23 discrete receptors [13]. Moreover, existing studies of chemokines in diabetic eye disease found conflicting results [14, 15]. Conventional meta-analysis can only focus on a single biomarker of a single disease, and cannot identify the most important biomarker from a set of two or more biomarkers of a single disease [16]. Therefore, it is necessary to conduct systematic, literature retrieval through extensive search terms utilizing as many possible variant chemokine names as possible, and using high-quality, network meta-analysis to quantitatively compare different chemokines and rank them according to the unified effect index [17]. We hope this study will contribute to a better understanding of both the signaling pathways of the chemokines in the pathophysiological process, and more reliable therapeutic targets for diabetic eye disease.

## Materials and methods

### Search strategy and selection criteria

Two independent reviewers (XP and AK) searched and selected relevant studies from the databases of PubMed, Web of Science, Embase and Cochrane Library from inception to Sep 1, 2023 without language restrictions. There is a great need to standardize the naming of chemokines into a unified format. Based on both historical and current chemokine, and various subgroups in diabetic eye disease names, the search algorithm for each electronic database was designed and adjusted by experienced librarians. Boolean operators, truncation and wildcards were widely used to allow for variant chemokine names and various subgroups in diabetic eye disease names (Additional file 1: Table S1).

### Study selection

Studies were selected for network meta-analysis based on the following inclusion criteria: (1) original research with reported data from cross-sectional or case–control studies, or baseline data from longitudinal studies; (2) studies with reported methods for diagnosing diabetic eye disease; (3) studies with measured blood, serum, vitreous, tears, aqueous humor, or ocular fibrovascular membranes chemokine concentrations in diabetic eye disease, control subjects, and mean and standard deviation (SD) of the measurements; or (4) these data could be obtained from correspondence authors. Studies were excluded based on the following exclusion criteria: (1) letters, opinions, comments or case reports where diabetic eye disease subjects had a comorbidity with other ophthalmic diseases; (2) in vitro and animal studies of diabetic eye disease; (3) studies where the measuring of chemokine concentrations was pharmacologically challenged before their measurements, and not basal-immune active.

### Data extraction

Two reviewers (XP and AK) independently extracted relevant data from the eligible studies used a custom, data-extraction template. EpiData (version 3.0) and EndNote (version X 9.2) were used to remove duplicate data. Relevant data were stored in Excel spreadsheets (version Microsoft Excel 2013). The following characteristics data were extracted: (1) last name of the first author; (2) publication year; (3) country; (4) subject characteristics such as age (mean ± SD), sex, duration of diabetes (mean ± SD), fasting plasma glucose (FPG) levels, and hemoglobin A1c (HbA1c); (5) sample characteristics such as material of sample and chemokines concentrations (mean ± SD) for each group of diabetic eye disease and control, we also used Engauge data software (version 4.1) to extracted chemokines concentrations (mean ± SD) when data were presented only in graphical format; and (6) chemokines sample-test methods.

### Quality assessment

The detailed protocol registration number is CRD42019148305. Moreover, this study was done according to the Preferred Reporting Items for Systematic Reviews and Meta-Analyses (PRISMA), and Cochrane Handbook guidelines [18]. To assess the risk of bias and quality of the eligible studies, two reviewers (XP and AK) adapted the Newcastle–Ottawa Quality Assessment Scale (NOS) for case–control studies as recommended by the PRISMA and Cochrane Handbook guidelines. Discrepancies between the two independent reviewers were resolved by involving the correspondence (AK). Each eligible study was evaluated based on the NOS Scale. According to the PRISMA guidelines, studies were graded as high, moderate or low quality based on the scores 7–9, 4–6 and 0–3, respectively.

### Data synthesis and statistical analysis

To be conservative, standardized mean difference (SMD) and 95% confidence intervals (CIs) with Random-Effect Model were used in the meta-analysis within the R software (version 3.5.2) meta package [19]. The SMD (95% CIs) of chemokines concentrations (calculated with Cohen’s d) between controls and cases were used to calculate the effect size of each study [20]. Furthermore, the random-effect model with a restricted, maximum-likelihood estimator was used to synthesize the effect size across studies, given the heterogeneity of material (e.g., vitreous and aqueous humor, etc.) of chemokines [21].

Subgroup analysis was conducted with respect to the sampling specimens used in the study, the chemokines-detection methods, and the sex, age and duration of diabetes of study participants. This was done to explore whether different subgroups had influence on the overall results. Statistical heterogeneity among studies was measured by Cochran’s Q-statistic and quantified by I^2^ statistic for statistical variation across studies [22]. Additional sensitivity analyses were performed for overall outcome by leave-one-out method. The publication bias was assessed by Egger Funnel Plots and Egger Weighted Regression Test [23].

After meta-analyses were completed, transitivity hypothesis and local inconsistency were evaluated before applying network meta-analysis within Stata (version 15.0) using the network package [16]. Random-Effect Model with Bayesian Framework Meta-analysis was performed to compare different chemokines. The pooled estimates were summarized by Markov Chain Monte Carlo Method (a run-in period of 10,000 iterations was adequate to achieve convergence) with the execution of a consistency assumption and a further 50,000 iterations [24]. Furthermore, we used vague priors and average residual deviation to estimate the Goodness-of-fit [25]. The rank probabilities values were evaluated and obtained by Surface Under the Cumulative Ranking Curve (SUCRA) and rank plot. A higher SUCRA value suggests better association with diabetic eye disease [17]. Finally, all hypothesis tests in this study were two-sided with 5% significance level.

## Results

### Literature search

The utilization of a systematic search of four databases yielded a total of 5,003 studies, of which 2,957 were from Web of Science, 856 from Embase, 209 from Cochrane Library, and 981 from PubMed. After removal of duplicates (of the 3,749 abstracts reviewed), full-text review of 656 studies was conducted, of which 34 met criteria for this network meta-analysis (Additional file 1: Figure S1).

### Characteristics of eligible studies

The overall meta-analysis consisted of 33 different chemokines nodes comparisons including 11,465 subjects (6,559 cases and 4,906 controls). Based on the material of chemokines, 15 studies collected chemokines from aqueous humor, 15 from vitreous, two from blood, one from serum, and one from tears. Across countries: 8 studies were conducted in China, 8 in the Japan, 3 in Italy, 3 in Korea, 3 in Turkey, 2 in India, 2 in United States, and 1 in each of the following countries: Finland, Germany, Romania, Russia and Singapore. For methods of chemokines determination: 17 studies used ELISA whereas another 17 studies used Luminex. For study quality: NOS scores varied between 5 and 8, with 19 studies classified as high quality and 15 as moderate quality (Additional file 1: Table S2). Four subfamilies of chemokines are summarized in Additional file 1: Table S3 and Additional file 1: Table S4 which are: common name; systematic name; classification of chemokines and their receptors; and different distribution of chemokines in different immune cells (e.g., CD4 T cells, CD8 T cells, monocytes, mast cells, basophils, neutrophils, dendric cells, etc.). Overall, the inconsistency-test comparison was not significant in network meta-analysis (p = 0.58), and the assumptions for using consistency models were satisfied. However, in the node-splitting test, there was a significant difference between direct and indirect coefficients (CCL1-CCL4, CCL3-CCL4, CCL3-CCL5 and CXCL1-CXCL5), but no significant differences in node-splitting model were observed in other comparisons (Additional file 1: Table S5 and Additional file 1: Table S6).

### Main findings

**Fig. 1:**
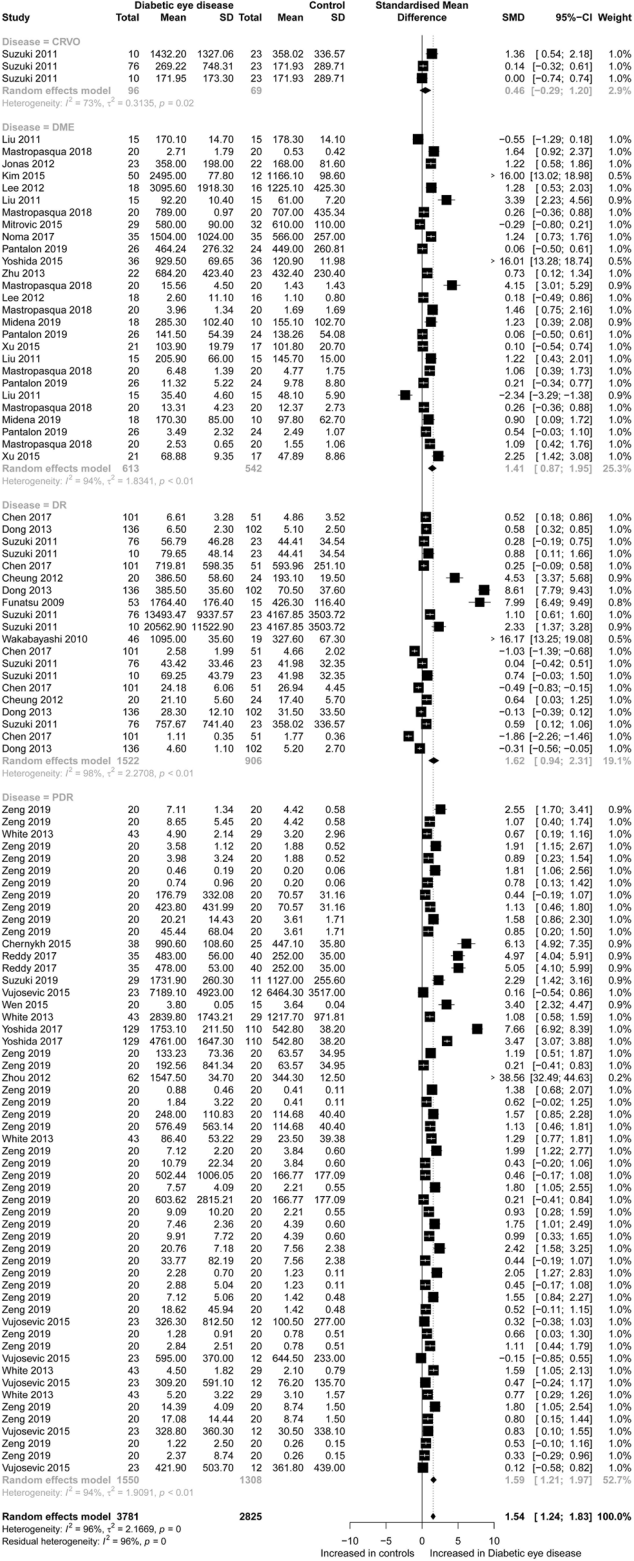
Forest plot of CC chemokines between diabetic eye disease patients and controls

**Fig. 2:**
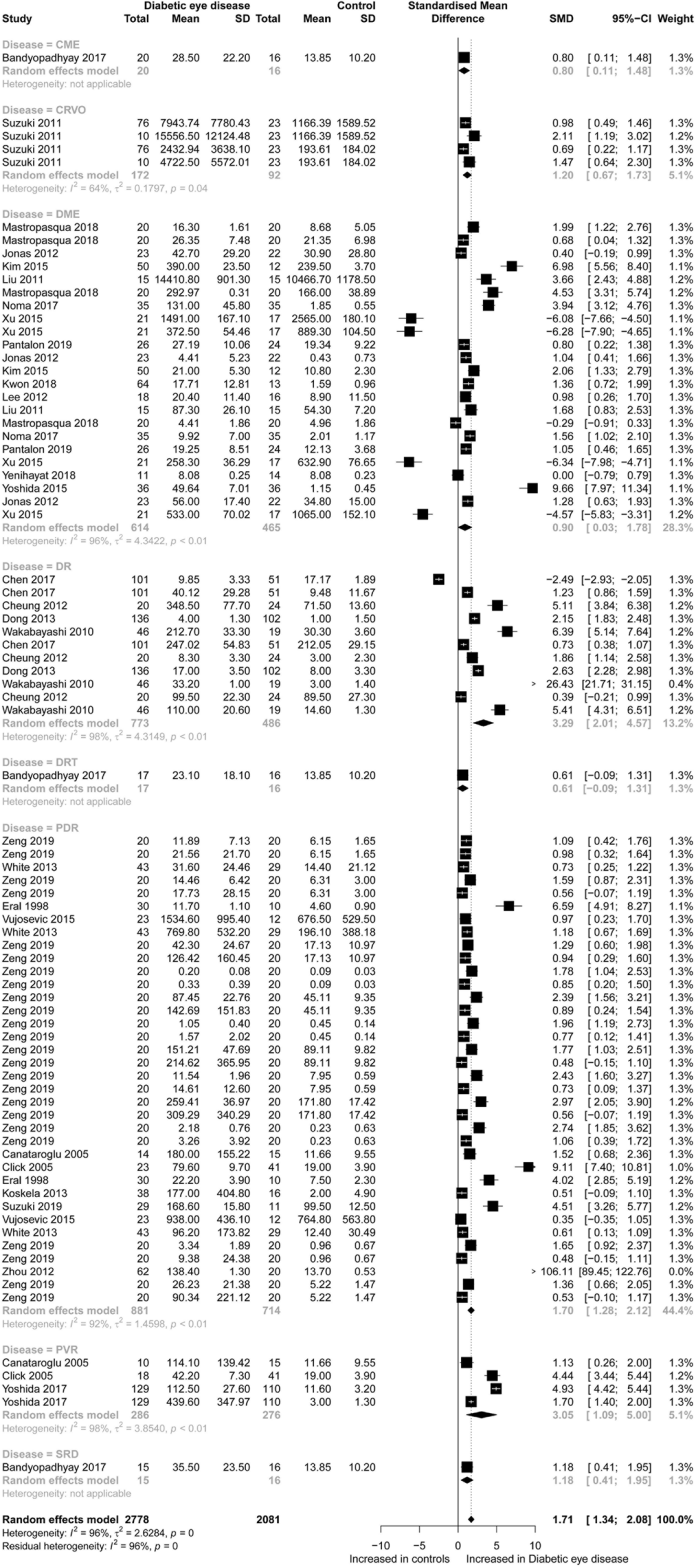
Forest plot of CXC chemokines between diabetic eye disease patients and controls

For CC chemokines, 3,781 diabetic eye disease patients and 2,825 controls were included in the meta-analysis. Concentrations of CC chemokines (SMD = 1.54; 95% CI 1.24 to 1.83; Fig. 1) were higher in the diabetic eye disease patients than in the controls. However, significant heterogeneity was observed among the included studies (I^2^ = 96%). Specifically, after subgroup analysis according to disease-type, the results of these subgroups (DME, DR and PDR) were still significant. For CXC chemokines, 2,778 diabetic eye disease patients and 2,081 controls were included in the meta-analysis. Concentrations of CXC chemokines (SMD = 1.71; 95% CI 1.34 to 2.08; Fig. 2) were higher in the diabetic eye disease patients than in the controls. Also, significant heterogeneity was observed between the included studies (I^2^ = 96%). Specifically, after subgroup analysis according to disease-type, the results of these subgroups (CME, CRVO, PVR, SRD, DME, DR and PDR) were still significant. Specifically, concentrations of CC chemokines (CCL1, CCL4, CCL7, CCL8, CCL11, CCL13, CCL15, CCL19, CCL2, CCL20, CCL21, CCL22, CCL23, CCL24, CCL25, CCL26 and CCL27) and CXC chemokines (CXCL2, CXCL5, CXCL6, CXCL8, CXCL9, CXCL10, CXCL11, CXCL12, CXCL13, CXCL16 and CX3CL1) were higher in the diabetic eye disease patients than in the controls. However, concentrations of chemokines (CCL3, CCL9 and CXCL1) retained no significance alone and concentrations of chemokines (CCL5 and CXCL4) were lower in the diabetic eye disease patients than in the controls (Additional file 1: Figure S2). Moreover, the sensitivity analysis indicated that any single study had little influence on the overall results.

### Subgroup analysis results

In subgroup analysis, there were significant differences between some subgroups (material of samples, chemokines detection methods, sex and diabetes duration), while no significant differences among other subgroups (race and age). Further details of subgroup analysis can be found in Table 1. There was a significant increase in aqueous humor, serum and vitreous chemokines between diabetic eye disease and control subjects—similar to that observed across studies where there was neither blood nor tears collected. In sex subgroup analysis, the chemokine-concentration-effect value (SMD) for males was significantly higher than for females. Moreover, comparisons of chemokines detection methods revealed the effect value (SMD) of ELISA was significantly higher than Luminex. For diabetes duration, subgroup analysis revealed a significant difference in the subgroup with diabetic duration of more than 10 years, but not in the subgroup lower than 10 years.

**Table 1:** Subgroup Analysis of chemokines in diabetic eye disease

Subgroup		SMD*	95% CI^**^		Heterogeneity		
					Q statistic	τ^2^	I^2^
Material	Aqueous humor	1.27	0.92	1.62	1708.91	1.90	96.30%
	Blood	− 2.93	− 5.11	− 0.76	228.14	8.19	97.40%
	Serum	5.01	4.34	5.67	0.01	0.00	0.00%
	Tears	1.15	− 0.52	2.82	99.59	4.12	95.00%
	Vitreous	2.04	1.74	2.34	2138.19	2.24	95.10%
Race	Caucasoid	1.27	0.91	1.62	356.53	1.00	90.50%
	Mongoloid	1.70	1.43	1.97	4303.26	2.71	96.50%
Sex	Female	1.20	0.92	1.48	1864.90	1.99	94.50%
	Male	1.98	1.61	2.35	2536.95	2.58	97.00%
	NR^***^	5.95	3.88	8.02	28.83	3.96	89.60%
Age (years)	< 60	1.71	1.43	1.99	2622.35	2.14	95.70%
	≥ 60	1.36	0.95	1.78	1957.92	2.81	96.50%
	NR	5.23	2.71	7.75	6.05	2.76	83.50%
Methods	ELISA^****^	2.22	1.58	2.86	2693.10	4.41	98.40%
	Luminex	1.44	1.24	1.65	1861.25	1.39	92.50%
Duration (years)	< 10	− 0.06	− 0.55	0.42	821.76	1.97	95.90%
	≥ 10	2.12	1.81	2.43	2768.26	2.54	96.00%
	NR	1.64	1.27	2.01	535.00	1.28	92.70%

^*^SMD, standardised mean difference

^**^CI, confidence interval

^***^NR, not report

^****^ELISA, Enzyme Linked Immunosorbent Assay

### Network meta-analysis results

**Fig. 3:**
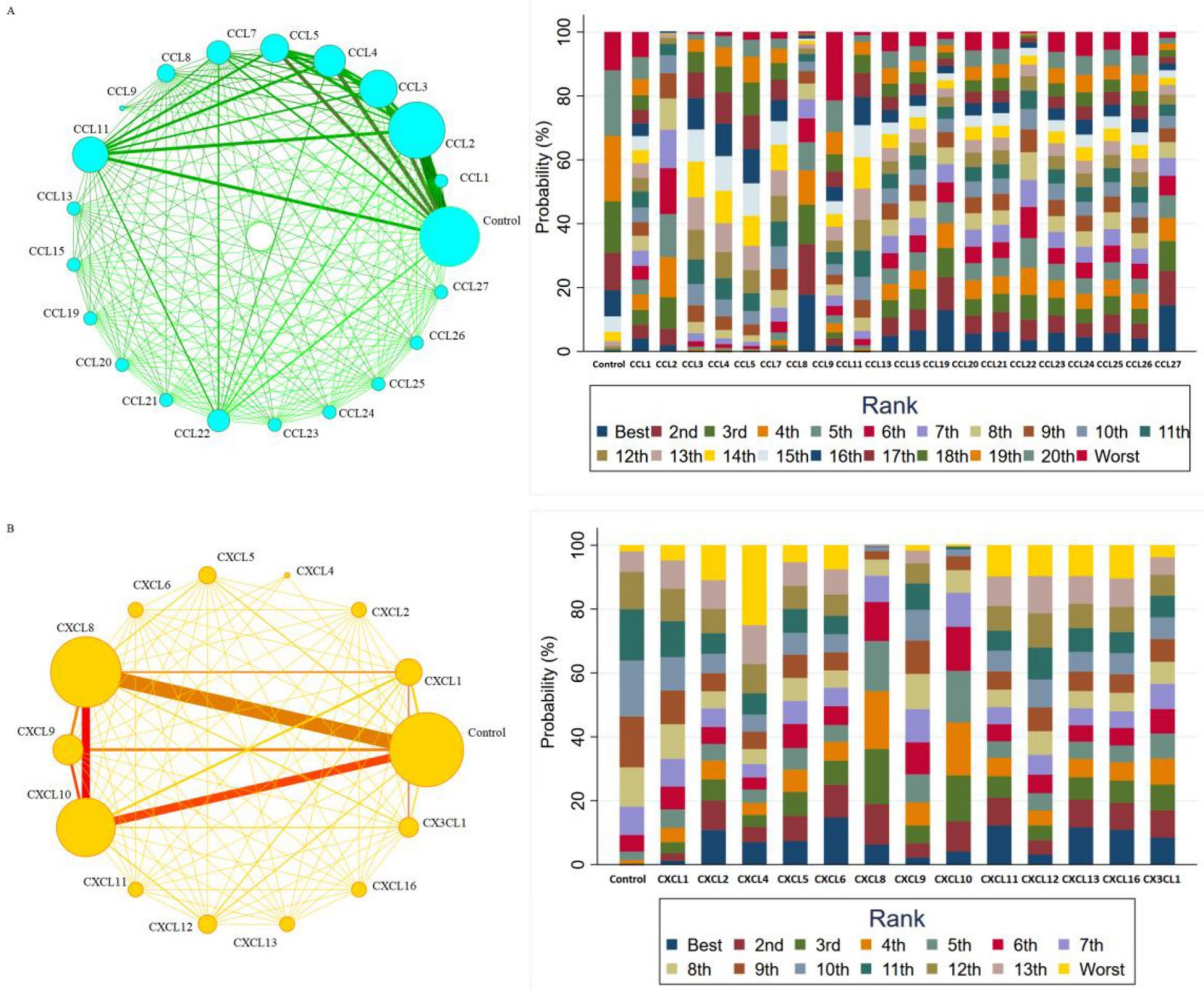
Network meta-analysis of CC chemokines (**A**) and CXC chemokines (**B**) comparisons for diabetic eye disease and rank probability of chemokines in diabetic eye disease for response rate in the network analysis

The network meta-analysis of CC and CXC chemokines consisted of 21 nodes and 14 nodes, respectively. Furthermore, each node involved a different chemokine in patients with diabetic eye disease and the controls (Fig. 3). Additional file 1: Table S7 and Additional file 1: Table S8 showed hierarchies of effect sizes on the basis of SUCRA rankings for CC and CXC chemokines. The top two, CCL8 and CCL2, each ranked as having the highest rank probabilities in CC chemokines network meta-analysis (SUCRA were 81.3% and 73.4%, respectively). Additionally, CXCL8 and CXCL10 were the top two in terms of rank probabilities in CXC chemokines network meta-analysis (SUCRA was 73.3% and 69.0%, respectively). Finally, the Egger’s Funnel Plots and Egger’s Test (t = 1.33, p = 0.12) for diabetic eye disease showed no evidence of asymmetry (Additional file 1: Figure S3).

## Discussion

To the best of our knowledge, this is the first network meta-analysis that evaluated the role of chemokines in diabetic eye disease. Previous studies have shown that various chemokines and their mediated immune microenvironment may be associated with diabetic eye disease [26, 27]. The challenge for these studies was that it was difficult to confirm this association in individual studies due to limited sample size. Therefore, this study was able to summarize available evidence on this association through network meta-analysis to rank relative importance of chemokines among various chemokines. Specifically, this network meta-analysis indicated that CC chemokines (CCL8 and CCL2) and CXC chemokines (CXCL8 and CXCL10) may discriminate between those with and without diabetic eye disease. Our results showed that the included studies mainly focused on retinopathy (DR, PDR and DME). Other diabetic eye diseases (PVR, CME, SRD, CRVO and DRT) included only a small number of studies and need to be interpreted with cautions. Therefore, the focus of this study was on the association between chemokines and retinopathy. Future studies need to further explore the role of chemokines in other diabetic eye diseases including PVR, CME, SRD, CRVO and DRT.

Although the mechanism by which hyperglycemia causes retinopathy progression is not fully understood, studies have shown that chemokines may be involved in multiple pathways of retinopathy, including nuclear-factor kappa-light-chain-enhancer of activated B cells (NF-κB) pathway, oxidative stress, dysregulation of reactive oxygen species (ROS) and nitric oxide synthase (NOS), activation of signal transducers and activators of transcription proteins (STATs), and formation of advanced glycation end-products (AGEs) [26–28]. These pathways could lead to osmotic vascular damage and retinopathy by activation of mitogen activated protein kinases (MAPKs), inducing the production of ICAM-1 and VCAM-1, and the breakdown of the vascular junction proteins [11, 29].

Pre-clinical studies have demonstrated the key role of chemokines in the pathogenesis of DR and PDR [30]. DR is an early stage of retinopathy characterized by blood-retina barrier breakdown and retinal inflammation. PDR is an advanced retinal inflammation stage of DR as the consequence of vascular function is impaired by capillary non-perfusion, occlusion and degeneration. Thus, vision loss or even blindness may be caused by DME, hemorrhaging and subsequent retinal detachment [28].

Studies have shown that CCL2, CCL8 and CXCL10 belong to the monocyte chemoattractants while CXCL8 belongs to the neutrophil-specific chemoattractants [31, 32]. During inflammation, these chemokines were in response to an inflammatory stimulus in the immune microenvironment of the DR and activated by recruiting monocyte and neutrophil to the site of inflammation [32]. Furthermore, experimental studies have shown that microglia, monocytes and neutrophils were attracted by chemokines and then scattered throughout the retina, forming an immune-microenvironment network [33, 34]. In this network, a large number of neutrophils and monocytes rapidly activate to attach to the vessel wall and block blood flow. This could lead to capillary occlusion, dropout and intra-retinal neovascularization, and injure vascular cells [35]. In the inflammatory response, inflammatory cytokines [such as tumor necrosis factor-α (TNF-α), ROS and NOS] initiated and perpetuated the immune cascade which rapidly activated their release. This further induces retinal and choroidal vessels microvascular injury [36]. In addition to participating in inflammatory reaction, neutrophils were also related to the upregulation of ICAM-1, which could lead to the closure of capillaries and further occurrence of DR [37]. These findings are consistent with our results.

During the development of DR to PDR, TNF-α could employ the nicotinamide adenine dinucleotide phosphate (NADPH) oxidase pathway to induce expression of CCL2 [38]. CCL2 and its mediated leukocyte recruitment could induce blood-retinal barrier (BRB) breakdown and microvascular cell death, leading to microvascular leakage and degeneration [39]. Moreover, a mechanism study has shown CCL2-induced, endothelial-barrier breakdown through multiple pathways, including protein kinase C (PKC) α/ζ-mediated translocation and phosphorylation of tight junction proteins (occludin, ZO-1, ZO-2, claudin-5) [40]. This notion has been supported by in-vitro studies with high glucose-treated retinal cells and vivo studies with diabetic models of ischemic retinopathy. Specifically, NADPH oxidase blockers significantly inhibit CCL2 production, NOS formation, and NF-κB activation, which could further prevent vascular leakage and pathological neovascularization [38, 41, 42].

Moreover, studies have shown that CXCL8 exacerbated immune cascade, caused phosphatidylinositol 3-kinases/Rac/p21, and activated a kinase-signaling axis. Together, this induced VE-cadherin internalization, thus leading to increased endothelial permeability [43]. CXCL8 was also found to recruit neutrophils and T-cells by binding to several different cell surface receptors including CXCR1 and CXCR2. Additionally, the interaction between leukocytes and vascular cells released matrix metallopeptidase (MMPs) and angiogenic factors leading to BRB breakdown, capillary degeneration, subsequent vessel leakage and induction of pathological neovascularization [44]. In general, these pathophysiological changes were reflected in clinical manifestations. The capillary degeneration and subsequent vessel leakage of PDR may induce DME and may cause vision loss. Furthermore, fragile pathological neovascularization could induce hemorrhaging and subsequent retinal detachment, which may lead to severe vision loss or even blindness [30].

Although these findings indicate that CC and CXC chemokines were associated with diabetic eye disease patients, high heterogeneity was observed. Subgroup analysis indicated that sample specimens, chemokines-detection methods and duration of diabetes might explain high heterogeneity. Subgroup analysis by sample specimens indicated that studies using aqueous humor, serum and vitreous chemokines were consistent with overall results. However, studies using blood and tears chemokines yielded inconsistent results. These findings suggested that blood and tears chemokines may induce instability.

**Fig. 4:**
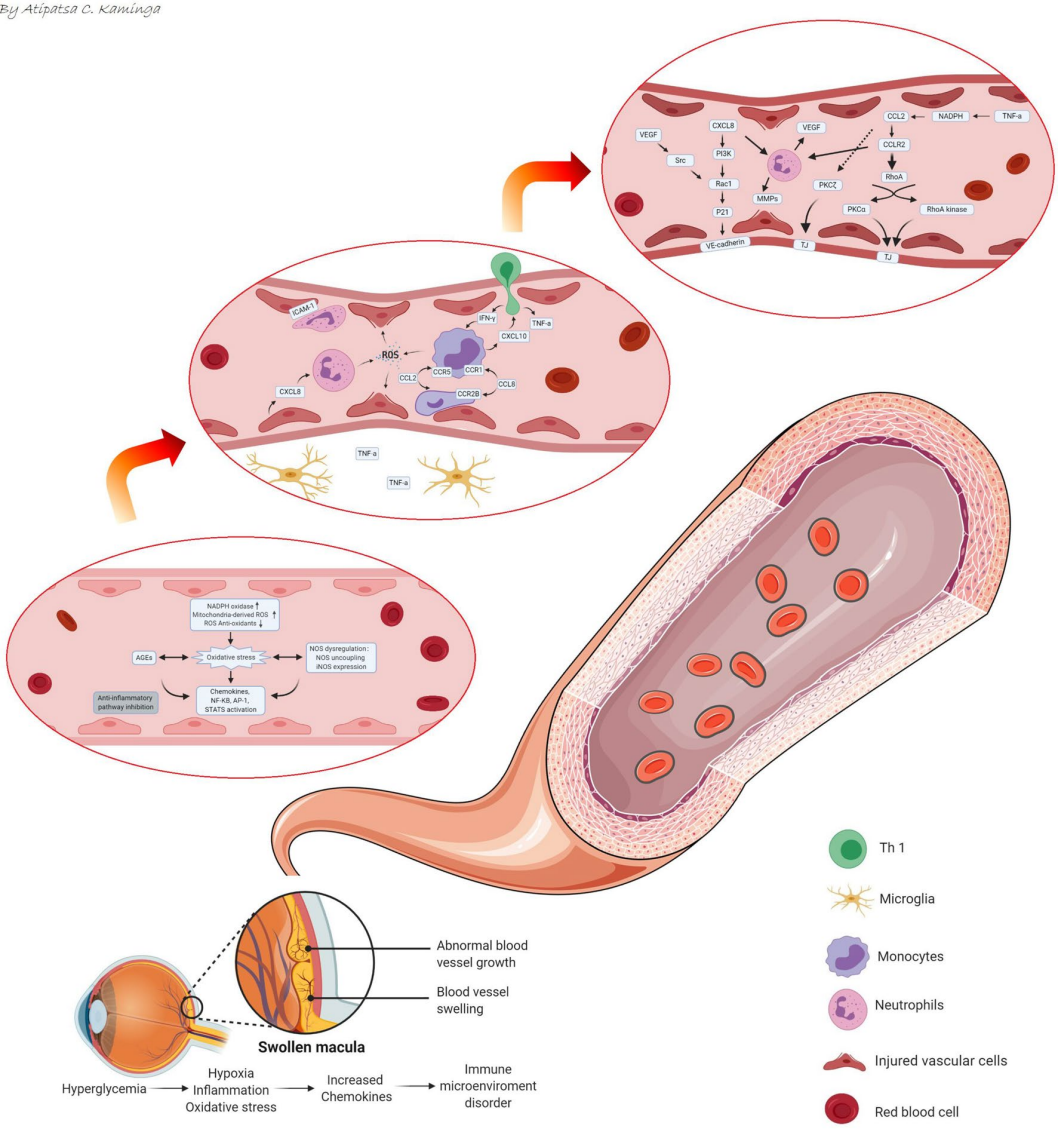
The complicated chemokines network in the microenvironment of diabetic eye disease

In sex subgroup analysis, the effect of chemokines in males was significantly higher than in females. This finding supports previous findings that males are at increased risk of developing diabetic retinopathy than females [45, 46]. One mechanism underlying the sex-related difference in the development of diabetic retinopathy may be sex-specific genetic differences and epigenetic modifications in chemokine gene function [47–49]. Therefore, we hypothesized that the testosterone and high, glucose-trigger, epigenetic modifications further induce the high expression of chemokines in males [50]. This may also affect the development of diabetic retinopathy through pro-inflammatory effect. We also systematically summarized the complicated chemokines network in the microenvironment of diabetic retinal disease (Fig. 4).

Subgroup analysis by duration of diabetes showed significant correlation with the diabetic eye disease. In fact, some studies reported diabetes duration as a risk factor for development of diabetic eye disease and other diabetic microvascular complications [45, 51]. These findings suggest that sex and diabetes duration may play key roles in diabetic retinopathy stratification, personalized prevention and interventions in the future.

### Strengths and limitations

There are a number of strengths to this study. First, by using broad inclusion criteria, comprehensive search strategies, and multiple variant names and including multiple databases, our study could be considered the most comprehensive, large-sample-size, and meta-analysis. Next, the quality of this meta-analysis was high since low quality studies were excluded. Finally, high-quality, network, meta-analysis has opened an innovative avenue to explore new biomarkers and molecule targets.

As a primary limitation, only a few eligible studies were included in some chemokine analysis (e.g., CCL1, CCL9, CCL13, CCL15 and CXCL4), which may have led to biased results due to statistical instability. Second, most original studies focused on chemokines in DR, PDR and DME. The association between chemokines and other diabetic eye diseases was less known. Future research should focus on other diabetic eye diseases including PVR, CME, SRD, CRVO, and DRT to fill this gap. Third, in the original studies, some important confounders (such as smoking, drinking and body mass index) were not measured nor were adjusted made. Therefore, these confounders should be considered in future research. Last, the mechanism for different stages of initiated and maintained chemokine-induced, cascade reactions in DR is unknown. Data in this study were all from case–control or cross-sectional studies which could not be used for causal inference. Therefore, mechanistic and longitudinal studies are essential to help clarify the role that chemokines may play in longer-term inflammation models of diabetic eye diseases.

## Conclusion

In conclusion, this study has comprehensively clarified the previously complicated and confused results. In addition, a number of chemokines that may be altered in diabetic eye disease patients have been identified. Network meta-analysis revealed that CCL8, CCL2, CXCL8 and CXCL10 may be involved in key pathophysiological process of diabetic eye disease. These findings may open up new perspectives in diabetic eye disease for early-diagnostic biomarkers and novel strategies for molecule-targeted drugs.

### Supplementary Information

**Additional file 1: Table S1.** Electronic search strategies. **Figure S1. **Study selection flow chart. **Table S2. **Characteristics of included studies. **Table S3. **The classification of chemokines and their receptors. **Table S4. **The distribution—cell type of chemokines receptors. **Table S5. **Local inconsistency for the network of CC chemokines in diabetic eye disease group (node-splitting method). **Table S6. **Local inconsistency for the network of CXC chemokines in diabetic eye disease group (node-splitting method). **Figure S2. **Forest plot of chemokines between diabetic eye disease group patients and controls **Table S7**. Rank and SUCRA of the effect of different CC chemokines in diabetic eye disease **Table S8. **Rank and SUCRA of the effect of different CXC chemokines in diabetic eye disease. **Figure S3**. Egger funnel plots of diabetic eye disease patients compared to controls.

## Data Availability

No data was used for the research described in the article.

## Abbreviations

SMDs: Standardized mean differences
CIs: Confidence intervals
chemokines: Chemotactic cytokines
SD: Standard deviation
FPG: Fasting plasma glucose
HbA1c: Hemoglobin A1c
PRISMA: Systematic Reviews and Meta-Analyse
NOS: Newcastle–Ottawa Quality Assessment Scale
SUCRA: Surface Under the Cumulative Ranking Curve
NF-κB: Nuclear-factor kappa-light-chain-enhancer of activated B cells
ROS: Reactive oxygen species
NOS: Nitric oxide synthase
STATs: Signal transducers and activators of transcription proteins
AGEs: Advanced glycation end-products
MAPKs: Mitogen activated protein kinases
TNF-α: Tumor necrosis factor-α
NADPH: Nicotinamide adenine dinucleotide phosphate
BRB: Blood-retinal barrier
PKC: Protein kinase C
MMPs: Matrix metallopeptidase
