# Supplementary material for: Chemokines in diabetic eye disease
Source: Diabetol Metab Syndr. 2024 May 24;16:115. doi: 10.1186/s13098-024-01297-w (PMC11127334; doi:10.1186/s13098-024-01297-w)
Supplement: Supplementary file 1 — Additional file 1: Table S1. Electronic search strategies. Figure S1. Study selection flow chart. Table S2. Characteristics of included studies. Table S3. The classification of chemokines and their receptors. Table S4. The distribution—cell type of chemokines receptors. Table S5. Local inconsistency for the network of CC chemokines in diabetic eye disease group (node-splitting method). Table S6. Local inconsistency for the network of CXC chemokines in diabetic eye disease group (node-splitting method). Figure S2. Forest plot of chemokines between diabetic eye disease group patients and controls Table S7. Rank and SUCRA of the effect of different CC chemokines in diabetic eye disease Table S8. Rank and SUCRA of the effect of different CXC chemokines in diabetic eye disease. Figure S3. Egger funnel plots of diabetic eye disease patients compared to controls. [file 13098_2024_1297_MOESM1_ESM.docx]

**Additional Materials**

# Contents

**Table S1:** Electronic search strategies

**Figure S1:** Study selection flow chart

**Table S2:** Characteristics of included studies

**Table S3:** The classification of chemokines and their receptors

**Table S4:** The distribution—cell type of chemokines receptors

**Table S5:** Local inconsistency for the network of CC chemokines in diabetic eye disease group (node-splitting method)

**Table S6:** Local inconsistency for the network of CXC chemokines in diabetic eye disease group (node-splitting method)

**Figure S2:** Forest plot of chemokines between diabetic eye disease group patients and controls **Table S7**: Rank and SUCRA of the effect of different CC chemokines in diabetic eye disease **Table S8:** Rank and SUCRA of the effect of different CXC chemokines in diabetic eye disease

**Figure S3**: Egger funnel plots of diabetic eye disease patients compared to controls

# Appendices References

**Table S1: Electronic search strategies.**

| **Search** | **Query** |
| --- | --- |
| 1 | chemokine* |
| 2 | ccl1 or ccl2 or ccl3 or ccl4 or ccl5 or ccl6 or ccl7 or ccl8 or ccl9 or ccl10 or ccl11 or ccl12 or ccl13 or ccl14 or ccl15 or ccl16 or ccl17 or ccl18 or ccl19 or ccl20 or ccl21 or ccl22 or ccl23 or ccl24 or ccl25  or ccl26 or ccl27 or ccl28 |
| 3 | cxcl1 or cxcl2 or cxcl3 or cxcl4 or cxcl5 or cxcl6 or cxcl7 or cxcl8 or cxcl9 or cxcl10 or cxcl11 or  cxcl12 or cxcl13 or cxcl14 or cxcl15 or cxcl16 or cxcl17 |
| 4 | xcl1 or xcl2 |
| 5 | cx3cl1 |
| 6 | ccl or cxcl or xcl or cx3cl |
| 7 | scya1 or scya2 or scya3 or scya4 or scya5 or scya6 or scya7 or scya8 or scya9 or scya10 or scya11 or scya12 or scya13 or scya14 or scya15 or scya16 or scya17 or scya18 or scya19 or scya20 or scya21  or scya22 or scya23 or scya24 or scya25 or scya26 or scya27 or scya28 |
| 8 | scyb1 or scyb2 or scyb3 or scyb4 or scyb5 or scyb6 or scyb7 or scyb8 or scyb9 or scyb10 or scyb11  or scyb12 or scyb13 or scyb14 or scyb15 or scyb16 or scyb17 |
| 9 | scyc1 or scyc2 |
| 10 | sycd1 |
| 11 | scya or scyb or scyc or scyd |

| 12 | chemokine receptor* |
| --- | --- |
| 13 | ccr1 or ccr2 or ccr2b or ccr3 or ccr4 or ccr5 or ccr6 or ccr7 or ccr8 or ccr9 or ccr10 |
| 14 | cxcr1 or cxcr2 or cxcr3 or cxcr3b or cxcr4 or cxcr5 or cxcr6 or cxcr7 |
| 15 | xcr1 |
| 16 | cx3cr1 |
| 17 | ccr or cxcr or xcr or cx3cr |
| 18 | chemotactic cytokine* or chemokine* |
| 19 | i-309 or i309 or tca-3 or tca3 or sise |
| 20 | IL-8 or GCP-2 or CXCR1 or NAP-2 or ENA-78 or GROα or GROβ or GROγ or PF4 or IP-10 or MIG or I-TAC or SDF-1 or BCA-1 or SR-PSOX or BRAK or MCP-1 or MCP-4 or CCR2 or  MCP-3 or MCP-2 or MIP-1β or MIP-1α or CCR5 or RANTES or MPIF-1 or HCC-1 or HCC-2 or HCC-4 or Eotaxin or Eotaxin-2 or Eotaxin-3 or TARC or CCR4 or MDC or MIP-3α or ELC or CCR7 or SLC or I-309 or TECK or CTACK or MEC or PARC or Lymphotactin or XCR1 or  SCM-1β or Fractalkine or Chemerin |
| 21 | S1 OR S2 OR S3 OR S4 OR S5 OR S6 OR S7 OR S8 OR S9 OR S10 OR S11 OR S12 OR S13 OR  S14 OR S15 OR S16 OR S17 OR S18 OR S19 OR S20 |
| 22 | Vitreoretinopathy neovascular inflammatories OR Proliferative vitreoretinopathy OR Diabetic retinopathy OR Angiopathies, diabetic OR Angiopathy, diabetic OR Diabetic angiopathy OR Diabetic vascular diseases OR Diabetic vascular disease OR Vascular disease, diabetic OR Vascular  diseases, diabetic OR Diabetic vascular complications OR Diabetic vascular complication OR |

|  | Vascular complication, diabetic OR Vascular complications, diabetic OR Microangiopathy, diabetic  OR Diabetic microangiopathies OR Diabetic microangiopathy OR Microangiopathies, diabetic |
| --- | --- |
| 23 | Diffuse retinal thickening OR Cystoid macular edema OR Serous retinal detachment OR Edema, macular OR Macular edema, cystoid OR Edema, cystoid macular OR Proliferative diabetic retinopathy OR Proliferative vitreoretinopathy OR Proliferative vitreoretinopathies OR Vitreoretinopathies, proliferative OR Vitreoretinopathy neovascular inflammatory OR Inflammatories, vitreoretinopathy neovascular OR Inflammatory, vitreoretinopathy neovascular OR  Neovascular inflammatories, vitreoretinopathy OR Neovascular inflammatory, vitreoretinopathy |
| 24 | S22 OR S23 |
| 25 | S21 AND S24 |

**2,957 of Web of Science**

(TOPIC: ((((((((((((((((((((((((((((((((Diffuse retinal thickening OR Cystoid macular edema) OR Serous retinal detachment) OR Edema, macular) OR Macular edema, cystoid) OR Edema, cystoid macular) OR Proliferative diabetic retinopathy) OR Proliferative vitreoretinopathy) OR Proliferative vitreoretinopathies) OR Vitreoretinopathies, proliferative) OR Vitreoretinopathy neovascular inflammatory) OR Inflammatories, vitreoretinopathy neovascular) OR Inflammatory, vitreoretinopathy neovascular) OR Neovascular inflammatories, vitreoretinopathy) OR Neovascular inflammatory, vitreoretinopathy) OR Vitreoretinopathy neovascular inflammatories) OR Proliferative vitreoretinopathy) OR Diabetic retinopathy) OR Angiopathies, diabetic) OR Angiopathy, diabetic) OR Diabetic angiopathy) OR Diabetic vascular diseases) OR Diabetic vascular disease) OR Vascular disease, diabetic) OR Vascular diseases, diabetic) OR Diabetic vascular complications) OR Diabetic vascular complication) OR Vascular complication, diabetic) OR Vascular complications, diabetic) OR Microangiopathy, diabetic) OR Diabetic microangiopathies) OR Diabetic microangiopathy) OR Microangiopathies, diabetic) AND TOPIC: ((((((((((((((((((((((((((((((((((((((((((((((((((((((((((((((((((((((((((((((((((((((((((((((((((((((((((((((((((((((

(((((((((((((((((((((((((((((((((((((((((((((((((((((((((((((((((((((((((((((((((((((((((((((((((((chemokine* OR

ccl1) OR ccl2) OR ccl3) OR ccl4) OR ccl5) OR ccl4) OR ccl7) OR ccl8) OR ccl4) OR cxcl10) OR ccl21) OR cclf2) OR cxcl13) OR ccl19) OR ccl17) OR ccl19) OR ccl17) OR ccl18) OR ccl19) OR ccl20) OR ccl21) OR ccl22) OR ccl23) OR ccl2f) OR ccl25) OR ccl26) OR ccl2f) OR ccl28) OR cxcl1) OR cxcl2) OR cxcl3) OR cxcr4) OR cxcl5) OR c2cl6) OR cxcl1) OR cxcl8) OR cxcl9) OR cxcl10) OR cxcl11) OR cxcl12) OR cxcl13) OR cxcl10) OR cxcl12) OR cxcl16) OR cxcl10) OR xclx) OR xch2) OR cx3cl1) OR ccl) OR cxcl) OR xcl) OR ch3cl) OR scca1) OR scca2) OR

scya2) OR scya2) OR scya2) OR scya2) OR scya2) OR scya2) OR scya2) OR scya17) OR scya17) OR scya17) OR scya17) OR scya17) OR scya17) OR scya17) OR scya11) OR scya17) OR scya17) OR scna20) OR scya11) OR scya20) OR scya20) OR scya20) OR scya20) OR scya20) OR scya17) OR scya20) OR scyl1) OR scya2) OR scya3) OR scya4) OR scya5) OR scyb5) OR scya7) OR scyb5) OR sc5b9) OR scyb11) OR scya11) OR scyb10) OR scyb10) OR scyb10) OR scyb10) OR scyb10) OR scya17) OR scyl1) OR sc2c2) OR sycp1) OR soya) OR scab) OR sclc) OR scid) OR chemokine receptor*) OR ccr1) OR ccr2) OR chr2b) OR ccr3) OR ccr4) OR ccr5) OR ccr6) OR ccr7) OR ccr8) OR ccr9) OR cor10) OR cxcr1) OR cxcr2) OR cxcr3) OR cxcr4b) OR cxcr4) OR cxcr5) OR cxcr4) OR cxcr4) OR xcr1) OR cx3cr1) OR ccr) OR cxcr) OR xcr) OR cx3cr1) OR chemotactic cytokine*) OR chemokine*) OR i-309) OR i300) OR tca-3) OR tcam) OR side) OR IL-8) OR GCP-2) OR CXCR1) OR NAP-2) OR ENA-78) OR GROα) OR GROβ) OR GROγ) OR PF4) OR IP-10) OR MIG) OR I-TAC) OR SDF-1) OR BCA-1) OR SR-PSOX) OR BRAK) OR MCP-1) OR MCP-4) OR CCR2) OR MCP-3) OR MCP-2) OR MIP-1β) OR MIP-1α) OR CCR5) OR RANTES) OR MPIF-1) OR HCC-1) OR HCC-2) OR HCC-4) OR Eotaxin) OR Eotaxin-2) OR Eotaxin-3) OR TARC) OR CCR4) OR MDC) OR MIP-3α) OR ELC) OR CCR7) OR SLC) OR I-309) OR TECK) OR CTACK) OR MEC) OR PARC) OR Lymphotactin) OR XCR1) OR

SCM-1β) OR Fractalkine) OR Chemerin) OR Limphotactin) OR SCM-1) OR C-10) OR Mrp-1) OR MIP-1γ) OR MRP2) OR Eotaxin-1) OR MCP-5) OR Leukotactin-1) OR MIP-5) OR LEC) OR NCC-4) OR MTN1) OR MIP-4) OR AMAC1) OR ELC) OR MIP-3β) OR LARC) OR MIP-3) OR 6Ckine) OR MPIF-2) OR Eotaxin-2) OR MIP-4α) OR CTAK) OR MGSA) OR MIP-2α) OR MIP-2β) OR PF-4) OR BLC) OR Lungkine) OR SRPSOX))

# 856 of Embase

('diffuse retinal thickening':ab,ti OR 'cystoid macular edema':ab,ti OR 'serous retinal detachment':ab,ti OR 'edema, macular':ab,ti OR 'macular edema, cystoid':ab,ti OR 'edema, cystoid macular':ab,ti OR 'proliferative diabetic retinopathy':ab,ti OR 'proliferative vitreoretinopathies':ab,ti OR 'vitreoretinopathies, proliferative':ab,ti OR 'vitreoretinopathy neovascular inflammatory':ab,ti OR 'inflammatories, vitreoretinopathy neovascular':ab,ti OR 'inflammatory, vitreoretinopathy neovascular':ab,ti OR 'neovascular inflammatories, vitreoretinopathy':ab,ti OR 'neovascular inflammatory, vitreoretinopathy':ab,ti OR 'vitreoretinopathy neovascular inflammatories':ab,ti OR 'proliferative vitreoretinopathy':ab,ti OR 'diabetic retinopathy':ab,ti OR 'angiopathies, diabetic':ab,ti OR 'angiopathy, diabetic':ab,ti OR 'diabetic angiopathy':ab,ti OR 'diabetic vascular diseases':ab,ti OR 'diabetic vascular disease':ab,ti OR 'vascular disease, diabetic':ab,ti OR 'vascular diseases, diabetic':ab,ti OR 'diabetic vascular complications':ab,ti OR 'diabetic vascular complication':ab,ti OR 'vascular complication, diabetic':ab,ti OR 'vascular complications, diabetic':ab,ti OR 'microangiopathy, diabetic':ab,ti OR 'diabetic microangiopathies':ab,ti OR 'diabetic microangiopathy':ab,ti OR 'microangiopathies, diabetic':ab,ti) AND (ccl1:ab,ti OR ccl2:ab,ti OR ccl3:ab,ti OR ccl4:ab,ti OR ccl5:ab,ti OR ccl6:ab,ti OR ccl7:ab,ti OR ccl8:ab,ti OR ccl9:ab,ti OR ccl10:ab,ti OR ccl11:ab,ti OR ccl12:ab,ti OR ccl13:ab,ti OR ccl14:ab,ti OR ccl15:ab,ti OR ccl16:ab,ti OR ccl17:ab,ti OR ccl18:ab,ti OR ccl19:ab,ti OR ccl20:ab,ti OR ccl21:ab,ti OR ccl22:ab,ti OR ccl23:ab,ti OR ccl24:ab,ti OR ccl25:ab,ti OR ccl26:ab,ti OR ccl27:ab,ti OR ccl28:ab,ti OR cxcl1:ab,ti OR cxcl2:ab,ti OR cxcl3:ab,ti OR cxcl4:ab,ti OR cxcl5:ab,ti OR cxcl6:ab,ti OR cxcl7:ab,ti OR cxcl8:ab,ti OR cxcl9:ab,ti OR cxcl10:ab,ti OR cxcl11:ab,ti OR cxcl12:ab,ti OR cxcl13:ab,ti OR cxcl14:ab,ti OR

cxcl15:ab,ti OR cxcl16:ab,ti OR cxcl17:ab,ti OR xcl1:ab,ti OR xcl2:ab,ti OR cx3cl1:ab,ti OR ccl:ab,ti OR cxcl:ab,ti OR xcl:ab,ti OR cx3cl:ab,ti OR scya1:ab,ti OR scya2:ab,ti OR scya3:ab,ti OR scya4:ab,ti OR scya5:ab,ti OR scya6:ab,ti OR scya7:ab,ti OR scya8:ab,ti OR scya9:ab,ti OR scya10:ab,ti OR scya11:ab,ti OR scya12:ab,ti OR scya13:ab,ti OR scya14:ab,ti OR scya15:ab,ti OR scya16:ab,ti OR scya17:ab,ti OR scya18:ab,ti OR scya19:ab,ti OR scya20:ab,ti OR scya21:ab,ti OR scya22:ab,ti OR scya23:ab,ti OR scya24:ab,ti OR scya25:ab,ti OR scya26:ab,ti OR scya27:ab,ti OR scya28:ab,ti OR scyb1:ab,ti OR scyb2:ab,ti OR scyb3:ab,ti OR scyb4:ab,ti OR scyb5:ab,ti OR scyb6:ab,ti OR scyb7:ab,ti OR scyb8:ab,ti OR scyb9:ab,ti OR scyb10:ab,ti OR scyb11:ab,ti OR scyb12:ab,ti OR scyb13:ab,ti OR scyb14:ab,ti OR scyb15:ab,ti OR scyb16:ab,ti OR scyb17:ab,ti OR scyc1:ab,ti OR scyc2:ab,ti OR sycd1:ab,ti OR scya:ab,ti OR scyb:ab,ti OR scyc:ab,ti OR scyd:ab,ti OR 'chemokine receptor*':ab,ti OR ccr1:ab,ti OR ccr2b:ab,ti OR ccr3:ab,ti OR ccr6:ab,ti OR ccr8:ab,ti OR ccr9:ab,ti OR ccr10:ab,ti OR cxcr2:ab,ti OR cxcr3:ab,ti OR cxcr3b:ab,ti OR cxcr4:ab,ti OR cxcr5:ab,ti OR cxcr6:ab,ti OR cxcr7:ab,ti OR cx3cr1:ab,ti OR ccr:ab,ti OR cxcr:ab,ti OR xcr:ab,ti OR cx3cr:ab,ti OR 'chemotactic cytokine*':ab,ti OR chemokine*:ab,ti OR i309:ab,ti OR 'tca 3':ab,ti OR tca3:ab,ti OR sise:ab,ti OR 'il 8':ab,ti OR 'gcp 2':ab,ti OR cxcr1:ab,ti OR 'nap 2':ab,ti OR 'ena 78':ab,ti OR groα:ab,ti OR groβ:ab,ti OR groγ:ab,ti OR pf4:ab,ti OR 'ip 10':ab,ti OR mig:ab,ti OR 'i tac':ab,ti OR 'sdf 1':ab,ti OR 'bca 1':ab,ti OR 'sr psox':ab,ti OR brak:ab,ti OR 'mcp 1':ab,ti OR 'mcp 4':ab,ti OR ccr2:ab,ti OR 'mcp 3':ab,ti OR 'mcp 2':ab,ti OR 'mip 1β':ab,ti OR 'mip 1α':ab,ti OR ccr5:ab,ti OR rantes:ab,ti OR 'mpif 1':ab,ti OR 'hcc 1':ab,ti OR 'hcc 2':ab,ti OR 'hcc 4':ab,ti OR eotaxin:ab,ti OR 'eotaxin 3':ab,ti OR tarc:ab,ti OR ccr4:ab,ti OR mdc:ab,ti OR 'mip 3α':ab,ti OR ccr7:ab,ti OR slc:ab,ti OR 'i 309':ab,ti OR teck:ab,ti OR ctack:ab,ti OR mec:ab,ti OR parc:ab,ti OR lymphotactin:ab,ti OR xcr1:ab,ti OR 'scm 1β':ab,ti

OR fractalkine:ab,ti OR chemerin:ab,ti OR limphotactin:ab,ti OR 'scm 1':ab,ti OR 'c 10':ab,ti OR 'mrp 1':ab,ti OR 'mip 1γ':ab,ti OR mrp2:ab,ti OR 'eotaxin 1':ab,ti OR 'mcp 5':ab,ti OR 'leukotactin 1':ab,ti OR 'mip 5':ab,ti OR lec:ab,ti OR 'ncc 4':ab,ti OR mtn1:ab,ti OR 'mip 4':ab,ti OR amac1:ab,ti OR elc:ab,ti OR 'mip 3β':ab,ti OR larc:ab,ti OR 'mip 3':ab,ti OR 6ckine:ab,ti OR 'mpif 2':ab,ti OR 'eotaxin 2':ab,ti OR 'mip 4α':ab,ti OR ctak:ab,ti OR mgsa:ab,ti OR 'mip 2α':ab,ti OR 'mip 2β':ab,ti OR 'pf 4':ab,ti OR blc:ab,ti OR lungkine:ab,ti OR srpsox:ab,ti)

# 981 of Pubmed

(Diffuse retinal thickening[Title/Abstract] OR Cystoid macular edema[Title/Abstract] OR Serous retinal detachment[Title/Abstract] OR Edema, macular[Title/Abstract] OR Macular edema, cystoid[Title/Abstract] OR Edema, cystoid macular[Title/Abstract] OR Proliferative diabetic retinopathy[Title/Abstract] OR Proliferative vitreoretinopathy[Title/Abstract] OR Proliferative vitreoretinopathies[Title/Abstract] OR Vitreoretinopathies, proliferative[Title/Abstract] OR Vitreoretinopathy neovascular inflammatory[Title/Abstract] OR Inflammatories, vitreoretinopathy neovascular[Title/Abstract] OR Inflammatory, vitreoretinopathy neovascular[Title/Abstract] OR Neovascular inflammatories, vitreoretinopathy[Title/Abstract] OR Neovascular inflammatory, vitreoretinopathy[Title/Abstract] OR Vitreoretinopathy neovascular inflammatories[Title/Abstract] OR Proliferative vitreoretinopathy[Title/Abstract] OR Diabetic retinopathy[Title/Abstract] OR Angiopathies, diabetic[Title/Abstract] OR Angiopathy, diabetic[Title/Abstract] OR Diabetic angiopathy[Title/Abstract] OR Diabetic vascular diseases[Title/Abstract] OR Diabetic vascular disease[Title/Abstract] OR Vascular disease, diabetic[Title/Abstract] OR Vascular diseases, diabetic[Title/Abstract] OR Diabetic vascular complications[Title/Abstract] OR Diabetic vascular

complication[Title/Abstract] OR Vascular complication, diabetic[Title/Abstract] OR Vascular complications, diabetic[Title/Abstract] OR Microangiopathy, diabetic[Title/Abstract] OR Diabetic microangiopathies[Title/Abstract] OR Diabetic microangiopathy[Title/Abstract] OR Microangiopathies, diabetic[Title/Abstract]) AND (chemokine*[Title/Abstract] OR ccl1[Title/Abstract] OR ccl2[Title/Abstract] OR ccl3[Title/Abstract] OR ccl4[Title/Abstract] OR ccl5[Title/Abstract] OR ccl6[Title/Abstract] OR ccl7[Title/Abstract] OR ccl8[Title/Abstract] OR ccl9[Title/Abstract] OR ccl10[Title/Abstract] OR ccl11[Title/Abstract] OR ccl12[Title/Abstract] OR ccl13[Title/Abstract] OR ccl14[Title/Abstract] OR ccl15[Title/Abstract] OR ccl16[Title/Abstract] OR ccl17[Title/Abstract] OR ccl18[Title/Abstract] OR ccl19[Title/Abstract] OR ccl20[Title/Abstract] OR ccl21[Title/Abstract] OR ccl22[Title/Abstract] OR ccl23[Title/Abstract] OR ccl24[Title/Abstract] OR ccl25[Title/Abstract] OR ccl26[Title/Abstract] OR ccl27[Title/Abstract] OR ccl28[Title/Abstract] OR cxcl1[Title/Abstract] OR cxcl2[Title/Abstract] OR cxcl3[Title/Abstract] OR cxcl4[Title/Abstract] OR cxcl5[Title/Abstract] OR cxcl6[Title/Abstract] OR cxcl7[Title/Abstract] OR cxcl8[Title/Abstract] OR cxcl9[Title/Abstract] OR cxcl10[Title/Abstract] OR cxcl11[Title/Abstract] OR cxcl12[Title/Abstract] OR cxcl13[Title/Abstract] OR cxcl14[Title/Abstract] OR cxcl15[Title/Abstract] OR cxcl16[Title/Abstract] OR cxcl17[Title/Abstract] OR xcl1[Title/Abstract] OR xcl2[Title/Abstract] OR cx3cl1[Title/Abstract] OR ccl[Title/Abstract] OR cxcl[Title/Abstract] OR xcl[Title/Abstract] OR cx3cl[Title/Abstract] OR scya1[Title/Abstract] OR scya2[Title/Abstract] OR scya3[Title/Abstract] OR scya4[Title/Abstract] OR scya5[Title/Abstract] OR scya6[Title/Abstract] OR scya7[Title/Abstract] OR scya8[Title/Abstract] OR scya9[Title/Abstract] OR scya10[Title/Abstract] OR scya11[Title/Abstract] OR

| scya12[Title/Abstract] | OR | scya13[Title/Abstract] | OR | scya14[Title/Abstract] | OR |
| --- | --- | --- | --- | --- | --- |
| scya15[Title/Abstract] | OR | scya16[Title/Abstract] | OR | scya17[Title/Abstract] | OR |
| scya18[Title/Abstract] | OR | scya19[Title/Abstract] | OR | scya20[Title/Abstract] | OR |
| scya21[Title/Abstract] | OR | scya22[Title/Abstract] | OR | scya23[Title/Abstract] | OR |
| scya24[Title/Abstract] | OR | scya25[Title/Abstract] | OR | scya26[Title/Abstract] | OR |
| scya27[Title/Abstract] | OR | scya28[Title/Abstract] | OR | scyb1[Title/Abstract] | OR |

scyb2[Title/Abstract] OR scyb3[Title/Abstract] OR scyb4[Title/Abstract] OR scyb5[Title/Abstract] OR scyb6[Title/Abstract] OR scyb7[Title/Abstract] OR scyb8[Title/Abstract] OR scyb9[Title/Abstract] OR scyb10[Title/Abstract] OR scyb11[Title/Abstract] OR scyb12[Title/Abstract] OR scyb13[Title/Abstract] OR scyb14[Title/Abstract] OR scyb15[Title/Abstract] OR scyb16[Title/Abstract] OR scyb17[Title/Abstract] OR scyc1[Title/Abstract] OR scyc2[Title/Abstract] OR sycd1[Title/Abstract] OR scya[Title/Abstract] OR scyb[Title/Abstract] OR scyc[Title/Abstract] OR scyd[Title/Abstract] OR chemokine receptor*[Title/Abstract] OR ccr1[Title/Abstract] OR ccr2[Title/Abstract] OR ccr2b[Title/Abstract] OR ccr3[Title/Abstract] OR ccr4[Title/Abstract] OR ccr5[Title/Abstract] OR ccr6[Title/Abstract] OR ccr7[Title/Abstract] OR ccr8[Title/Abstract] OR ccr9[Title/Abstract] OR ccr10[Title/Abstract] OR cxcr1[Title/Abstract] OR cxcr2[Title/Abstract] OR cxcr3[Title/Abstract] OR cxcr3b[Title/Abstract] OR cxcr4[Title/Abstract] OR cxcr5[Title/Abstract] OR cxcr6[Title/Abstract] OR cxcr7[Title/Abstract] OR xcr1[Title/Abstract] OR cx3cr1[Title/Abstract] OR ccr[Title/Abstract] OR cxcr[Title/Abstract] OR xcr[Title/Abstract] OR cx3cr[Title/Abstract] OR chemotactic cytokine*[Title/Abstract] OR chemokine*[Title/Abstract] OR i-309[Title/Abstract] OR i309[Title/Abstract] OR tca-3[Title/Abstract] OR tca3[Title/Abstract] OR sise[Title/Abstract]

OR IL-8[Title/Abstract] OR GCP-2[Title/Abstract] OR CXCR1[Title/Abstract] OR NAP-2[Title/Abstract] OR ENA-78[Title/Abstract] OR GROα[Title/Abstract] OR GROβ[Title/Abstract] OR GROγ[Title/Abstract] OR PF4[Title/Abstract] OR IP-10[Title/Abstract] OR MIG[Title/Abstract] OR I-TAC[Title/Abstract] OR SDF-1[Title/Abstract] OR BCA-1[Title/Abstract] OR SR-PSOX[Title/Abstract] OR BRAK[Title/Abstract] OR MCP-1[Title/Abstract] OR MCP-4[Title/Abstract] OR CCR2[Title/Abstract] OR MCP-3[Title/Abstract] OR MCP-2[Title/Abstract] OR MIP-1β[Title/Abstract] OR MIP-1α[Title/Abstract] OR CCR5[Title/Abstract] OR RANTES[Title/Abstract] OR MPIF-1[Title/Abstract] OR HCC-1[Title/Abstract] OR HCC-2[Title/Abstract] OR HCC-4[Title/Abstract] OR Eotaxin[Title/Abstract] OR Eotaxin-2[Title/Abstract] OR Eotaxin-3[Title/Abstract] OR TARC[Title/Abstract] OR CCR4[Title/Abstract] OR MDC[Title/Abstract] OR MIP-3α[Title/Abstract] OR ELC[Title/Abstract] OR CCR7[Title/Abstract] OR SLC[Title/Abstract] OR I-309[Title/Abstract] OR TECK[Title/Abstract] OR CTACK[Title/Abstract] OR MEC[Title/Abstract] OR PARC[Title/Abstract] OR Lymphotactin[Title/Abstract] OR XCR1[Title/Abstract] OR SCM-1β[Title/Abstract] OR Fractalkine[Title/Abstract] OR Chemerin[Title/Abstract] OR Limphotactin[Title/Abstract] OR

| SCM-1[Title/Abstract] | OR | C-10[Title/Abstract] | OR Mrp-1[Title/Abstract] | OR |
| --- | --- | --- | --- | --- |
| MIP-1γ[Title/Abstract] | OR | MRP2[Title/Abstract] | OR Eotaxin-1[Title/Abstract] | OR |

MCP-5[Title/Abstract] OR Leukotactin-1[Title/Abstract] OR MIP-5[Title/Abstract] OR LEC[Title/Abstract] OR NCC-4[Title/Abstract] OR MTN1[Title/Abstract] OR MIP-4[Title/Abstract] OR AMAC1[Title/Abstract] OR ELC[Title/Abstract] OR MIP-3β[Title/Abstract] OR LARC[Title/Abstract] OR MIP-3[Title/Abstract] OR

6Ckine[Title/Abstract] OR MPIF-2[Title/Abstract] OR Eotaxin-2[Title/Abstract] OR MIP-4α[Title/Abstract] OR CTAK[Title/Abstract] OR MGSA[Title/Abstract] OR MIP-2α[Title/Abstract] OR MIP-2β[Title/Abstract] OR PF-4[Title/Abstract] OR BLC[Title/Abstract] OR Lungkine[Title/Abstract] OR SRPSOX[Title/Abstract])

# 209 of Cochrane Library

chemokine* or ccl1 or ccl2 or ccl3 or ccl4 or ccl5 or ccl6 or ccl7 or ccl8 or ccl9 or ccl10 or ccl11 or ccl12 or ccl13 or ccl14 or ccl15 or ccl16 or ccl17 or ccl18 or ccl19 or ccl20 or ccl21 or ccl22 or ccl23 or ccl24 or ccl25 or ccl26 or ccl27 or ccl28 or cxcl1 or cxcl2 or cxcl3 or cxcl4 or cxcl5 or cxcl6 or cxcl7 or cxcl8 or cxcl9 or cxcl10 or cxcl11 or cxcl12 or cxcl13 or cxcl14 or cxcl15 or cxcl16 or cxcl17 or xcl1 or xcl2 or cx3cl1 or ccl or cxcl or xcl or cx3cl or scya1 or scya2 or scya3 or scya4 or scya5 or scya6 or scya7 or scya8 or scya9 or scya10 or scya11 or scya12 or scya13 or scya14 or scya15 or scya16 or scya17 or scya18 or scya19 or scya20 or scya21 or scya22 or scya23 or scya24 or scya25 or scya26 or scya27 or scya28 or scyb1 or scyb2 or scyb3 or scyb4 or scyb5 or scyb6 or scyb7 or scyb8 or scyb9 or scyb10 or scyb11 or scyb12 or scyb13 or scyb14 or scyb15 or scyb16 or scyb17 or scyc1 or scyc2 or sycd1 or scya or scyb or scyc or scyd or chemokine receptor* or ccr1 or ccr2 or ccr2b or ccr3 or ccr4 or ccr5 or ccr6 or ccr7 or ccr8 or ccr9 or ccr10 or cxcr1 or cxcr2 or cxcr3 or cxcr3b or cxcr4 or cxcr5 or cxcr6 or cxcr7 or xcr1 or cx3cr1 or ccr or cxcr or xcr or cx3cr or chemotactic cytokine* or chemokine* or i-309 or i309 or tca-3 or tca3 or sise or IL-8 or GCP-2 or CXCR1 or NAP-2 or ENA-78 or GROα or GROβ or GROγ or PF4 or IP-10 or MIG or I-TAC or SDF-1 or BCA-1 or SR-PSOX or BRAK or MCP-1 or MCP-4

or CCR2 or MCP-3 or MCP-2 or MIP-1β or MIP-1α or CCR5 or RANTES or MPIF-1 or HCC-1

or HCC-2 or HCC-4 or Eotaxin or Eotaxin-2 or Eotaxin-3 or TARC or CCR4 or MDC or MIP-3α or ELC or CCR7 or SLC or I-309 or TECK or CTACK or MEC or PARC or Lymphotactin or XCR1 or SCM-1β or Fractalkine or Chemerin or Limphotactin or SCM-1 or C-10 or Mrp-1 or MIP-1γ or MRP2 or Eotaxin-1 or MCP-5 or Leukotactin-1 or MIP-5 or LEC or NCC-4 or MTN1 or MIP-4 or AMAC1 or ELC or MIP-3β or LARC or MIP-3 or 6Ckine or MPIF-2 or Eotaxin-2 or MIP-4α or CTAK or MGSA or MIP-2α or MIP-2β or PF-4 or BLC or Lungkine or SRPSOX in Title Abstract Keyword AND Diffuse retinal thickening OR Cystoid macular edema OR Serous retinal detachment OR Edema, macular OR Macular edema, cystoid OR Edema, cystoid macular OR Proliferative diabetic retinopathy OR Proliferative vitreoretinopathy OR Proliferative vitreoretinopathies OR Vitreoretinopathies, proliferative OR Vitreoretinopathy neovascular inflammatory OR Inflammatories, vitreoretinopathy neovascular OR Inflammatory, vitreoretinopathy neovascular OR Neovascular inflammatories, vitreoretinopathy OR Neovascular inflammatory, vitreoretinopathy OR Vitreoretinopathy neovascular inflammatories OR Proliferative vitreoretinopathy OR Diabetic retinopathy OR Angiopathies, diabetic OR Angiopathy, diabetic OR Diabetic angiopathy OR Diabetic vascular diseases OR Diabetic vascular disease OR Vascular disease, diabetic OR Vascular diseases, diabetic OR Diabetic vascular complications OR Diabetic vascular complication OR Vascular complication, diabetic OR Vascular complications, diabetic OR Microangiopathy, diabetic OR Diabetic microangiopathies OR Diabetic microangiopathy OR Microangiopathies, diabetic in Title Abstract Keyword

# Figure S1: Study selection flow chart.


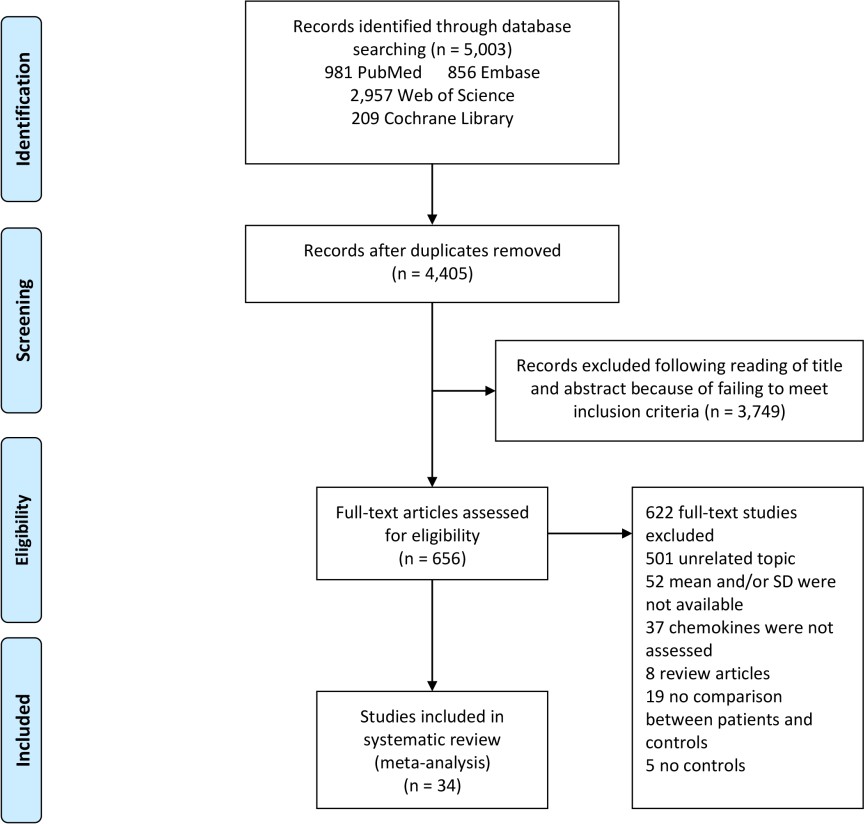


A flow chart demonstrating the selection process of articles included in the analysis as well as in the qualitative summary.

# Table S2: Characteristics of included studies.

| **Study** |  | **Control type** | **Types of sample** | **Country** | **Male gender, n (%)** | **Mean Age** | **Methods of chemokine** | **Duration of diabetes (years)** | **FPG (mmol/l)** | **HbA1c (%)** | **NOS** |
| --- | --- | --- | --- | --- | --- | --- | --- | --- | --- | --- | --- |
| Bandyopadhyay 2017 | [1] | HC | Aqueous humor | India | 28 (53.80%) | 61.2±6.5 | ELISA | 13.3±1.3 | NR | 7.3±0.5 | 7 |
| Canataroglu 2005 | [2] | HC | Vitreous | Turkey | 4 (28.57%) | 56.1±5.3 | ELISA | NR | NR | NR | 6 |
| Chen 2017 | [3] | HC | Aqueous humor | China | 24 (51.00%) | 58.8±8.9 | ELISA | 8.5±3.8 | 7.5±1.8 | 8.8±3.5 | 8 |
| Chernykh 2015 | [4] | HC | Vitreous | Russia | 16 (42.10%) | 50.5±3.2 | ELISA | 8±2.3 | NR | NR | 6 |
| Cheung 2012 | [5] | HC | Aqueous humor | Singapore | 18 (66.70%) | 67.4±10.7 | Luminex | 30.1±6.3 | 9.0±2.8 | NR | 7 |
| Click 2005 | [6] | HC | Vitreous | Turkey | NR | 55.9±2.4 | ELISA | NR | NR | NR | 5 |
| Dong 2013 | [7] | HC | Aqueous humor | China | 71 (52.2%) | 66.7±6.8 | ELISA | 18.1 | 8.6±2.6 | 7.8±2.5 | 8 |
| Eral 1998 | [8] | HC | Vitreous | USA | NR | NR | ELISA | NR | NR | NR | 5 |
| Funatsu 2009 | [9] | Diabetics without retinopathy control | Vitreous | Japan | 25 (47.17%) | 61.2±7.2 | ELISA | 18.8±5.1 | NR | 7.3±0.7 | 6 |
| White 2013 | [10] | Vitrectomy patients control | Vitreous | USA | 22 (51.16%) | 58.0±11.0 | Luminex | NR | NR | NR | 5 |
| Jonas 2012 | [11] | HC | Aqueous humor | Germany | 15 (65.22%) | 70.9±9.3 | Luminex | NR | NR | NR | 5 |
| Kim 2015 | [12] | HC | Aqueous humor | South Korea | 11 (57.89%) | 62.4±7.9 | Luminex | 11.2 | NR | 7.3±1.5 | 6 |
| Koskela 2013 | [13] | HC | Vitreous | Finland | 17 (44.70%) | 59.4±14.3 | ELISA | 24.7±12.3 | 10.5±4.2 | 8.5±1.5 | 8 |
| Kwon 2018 | [14] | Cataract control | Aqueous humor | Korea | 30 (46.89%) | 56.8±7.9 | Luminex | NR | NR | NR | 5 |
| Lee 2012 | [15] | HC | Aqueous humor | Korea | 9 (50.00%) | 54.8±12.3 | Luminex | NR | NR | NR | 5 |
| Liu 2011 | [16] | HC | Tears | China | 7 (46.66%) | 61.1±2.2 | Luminex | 9.0±1.2 | 8.2±0.8 | 9.9±0.7 | 8 |
| Mastropasqua 2018 | [17] | HC | Aqueous humor | Italy | 11 (55.00%) | 63.4±7.3 | Luminex | 12.8±7.1 | NR | 7.6±3.6 | 7 |
| Midena 2019 | [18] | HC | Aqueous humor | Italy | 10 (55.55%) | 63.0±8.7 | Luminex | 15.2±10.0 | NR | 7.4±2.6 | 7 |
| Mitrovic 2015 | [19] | HC | Blood | Japan | 55 (51.88%) | 65.2±9.6 | ELISA | NR | 7.6±2.6 | 6.9±1.5 | 7 |
| Noma 2017 | [20] | Cataract control | Aqueous humor | Japan | 24 (68.57%) | 64.3±10.3 | ELISA | 11.5±5.3 | NR | 7.9±1.4 | 7 |
| Pantalon 2019 | [21] | HC | Aqueous humor | Romania | 12 (46.15%) | 69.0±9.5 | Luminex | 9.6±1.8 | 7.7±3.5 | 7.1±1.4 | 8 |
| Reddy 2017 | [22] | HC | Serum samples | India | 19 (54.00%) | 44.0±10.0 | ELISA | 21.5±6.7 | 7.6±1.2 | 9.2±1.9 | 8 |
| Suzuki 2011 | [23] | Epiretinal membrane and macular hole control | Vitreous | Japan | 25 (44.64%) | 57.6±13.6 | Luminex | NR | NR | NR | 5 |
| Suzuki 2019 | [24] | Diabetics without retinopathy control | Vitreous | Japan | 21 (72.41%) | 59.3±10.1 | ELISA | 19.0±10.0 | NR | 7.1±1.3 | 7 |
| Vujosevic 2015 | [25] | HC | Aqueous humor | Italy | 6 (54.54%) | 68.9±11.4 | Luminex | 7.7±5.1 | NR | 7.8±0.6 | 7 |
| Wakabayashi 2010 | [26] | Nondiabetic macular disease | Vitreous | Japan | 29 (67.44%) | 59.6±11.1 | Luminex | 14.4±8.3 | NR | 6.9±1.4 | 7 |
| Wen 2015 | [27] | HC | Aqueous humor | China | 28 (54.90%) | 57.9±11.8 | Luminex | 12.0±2.4 | NR | NR | 6 |
| Xu 2015 | [28] | HC | Blood | China | 23 (46.00%) | 60.1±7.5 | ELISA | 8.9±5.2 | 8.9±4.2 | 9.5±2.5 | 8 |
| Yenihayat 2018 | [29] | HC | Vitreous | Turkey | 7 (63.60%) | 60.0±12.7 | ELISA | 16.6±6.0 | NR | NR | 6 |
| Yoshida 2015 | [30] | Self-control | Vitreous | Japan | 22 (66.60%) | 56.8±10.5 | ELISA | NR | NR | NR | 5 |
| Yoshida 2017 | [31] | HC | Vitreous | Japan | 6 (42.85%) | 55.4±14.4 | Luminex | 12.4±5.9 | NR | 7.8±1.6 | 7 |
| Zeng 2019 | [32] | HC | Vitreous | China | 9 (47.36%) | 55.6±7.6 | Luminex | 10.9±3.2 | NR | 7.2±1.0 | 7 |
| Zhou 2012 | [33] | HC | Vitreous | China | 30 (48.38%) | 62.8±8.9 | ELISA | 12.5±6.3 | NR | 8.2±3.8 | 7 |
| Zhu 2013 | [34] | Cataract control | Aqueous humor | China | 13 (59.09%) | 71.0±5.6 | Luminex | NR | NR | NR | 5 |

ELISA, enzyme linked immunosorbent assay; FPG, fasting plasma glucose; HbA1c, hemoglobin A1c; NR, not report; USA, United States of America.

# Table S3: The classification of chemokines and their receptors.

| **Subfamily of chemokine** | **Name of chemokine** | **Other names of chemokine** | **Receptors** |
| --- | --- | --- | --- |
| **C chemokines** | XCL1 | Limphotactin α, SCM-1α | XCR1 |
|  | XCL2 | Limphotactin β, SCM-1β | XCR1 |
| **CC chemokines** | CCL1 | I-309 | CCR8 |
|  | CCL2 | MCP-1 | CCR2 |
|  | CCL3 | MIP-1α | CCR1,CCR5 |
|  | CCL4 | MIP-1β | CCR5,CCR8 |
|  | CCL5 | RANTES | CCR1,CCR3,CCR5 |
|  | CCL6 | C-10,Mrp-1 | CCR1 |
|  | CCL7 | MCP-3 | CCR1,CCR2,CCR3 |
|  | CCL8 | MCP-2 | CCR1,CCR2,CCR3,CCR5,CCR8 |
|  | CCL9 | MIP-1γ,MRP2 | CCR1,CCR3 |
|  | CCL11 | Eotaxin-1 | CCR3,CCR5 |
|  | CCL12 | MCP-5 | CCR2 |
|  | CCL13 | MCP-4 | CCR1,CCR2，CCR3,CCR5 |
|  | CCL14 | HCC-1 | CCR1,CCR5 |
|  | CCL15 | HCC-2, Leukotactin-1, MIP-5 | CCR1,CCR3 |
|  | CCL16 | HCC-4, LEC, NCC-4, MTN1 | CCR1,CCR2,CCR5,CCR8 |
|  | CCL17 | TARC | CCR4 |
|  | CCL18 | MIP-4, AMAC1 | CCR8 |
|  | CCL19 | ELC,MIP-3β | CCR7 |
|  | CCL20 | LARC,MIP-3α | CCR6 |

|  | CCL21 | SLC,6Ckine | CCR7 |
| --- | --- | --- | --- |
|  | CCL22 | MDC | CCR4 |
|  | CCL23 | MPIF-1,MIP-3 | CCR1,CCR3 |
|  | CCL24 | MPIF-2,Eotaxin-2 | CCR3 |
|  | CCL25 | TECK | CCR9 |
|  | CCL26 | Eotaxin-3,MIP-4α | CCR3,CX3CR1 |
|  | CCL27 | CTAK | CCR10 |
|  | CCL28 | MEC | CCR3,CCR10 |
| **CXC chemokines** | CXCL1 | GROα,MGSA | CXCR2 |
|  | CXCL2 | GROβ,MIP-2α | CXCR2 |
|  | CXCL3 | GROγ,MIP-2β | CXCR2 |
|  | CXCL4 | PF-4 | CXCR3 |
|  | CXCL5 | ENA-78 | CXCR2 |
|  | CXCL6 | GCP-2 | CXCR1,CXCR2 |
|  | CXCL7 | NAP-2 | CXCR2 |
|  | CXCL8 | IL-8 | CXCR1,CXCR2 |
|  | CXCL9 | Mig | CXCR3 |
|  | CXCL10 | IP-10 | CXCR3 |
|  | CXCL11 | I-TAC | CXCR3,CXCR7 |
|  | CXCL12 | SDF-1 | CXCR4,CXCR7 |
|  | CXCL13 | BCA-1,BLC | CXCR5,CXCR3 |
|  | CXCL14 | BRAK | Unknown |
|  | CXCL15 | Lungkine | Unknown |

|  | CXCL16 | SRPSOX | CXCR6 |
| --- | --- | --- | --- |
| **CX3C chemokines** | CX3CL1 | Fractalkine | CX3CR1 |

**Table S4: The distribution—cell type of chemokines receptors.**

| **Receptors of Chemokine** | **Distribution—Cell Type** |
| --- | --- |
| **CXCR1** | Neutrophils, monocytes, mast cells, basophils, dendric cells, CD8 T cells,natural killer cells |
| **CXCR2** | Neutrophils, monocytes, mast cells, basophils, dendric cells, natural killer cells |
| **CXCR3** | Basophils, Th1 cells, CD8 T cells, natural killer cells, Treg cells |
| **CXCR4** | Widely expressed |
| **CXCR5** | Basophils, CD8 T cells |
| **CXCR6** | Th1 cells, Th17 cells, natural killer cells, plasma cells |
| **CCR2** | Monocytes, microglia, neutrophil, macrophages, Th1 cells, basophil, natural killer cells |
| **CCR5** | Dendric cells, monocytes, macrophages, natural killer cells, Th1 cells, TH17 cells, |
| **CCR1** | Neutrophils, monocytes, macrophages, Th1 cells, basophils, dendric cells |
| **CCR3** | Eosinophils, basophils, Th2 cells, mast cells, dendric cells |
| **CCR4** | Th2 cells, Th17 cells, Treg cells, monocytes, basophils, CD4 & CD8 T cells |
| **CCR6** | Th17 cells, natural killer cells, Treg cells |
| **CCR7** | Dendric cells (mature), T cells, basophils |
| **CCR8** | Dendirc cells, monocytes, macrophages, Th2 cells, Treg cells |
| **CCR9** | Basophils, dendric cells |
| **CCR10** | T cells, IgA+ plasma cells |
| **XCR1** | Dendric cells |
| **CX3CR1** | Monocytes, macrophages, Th1 cells, dendric cells, natural killer cells |

**Table S5: Local inconsistency for the network of CC chemokines in diabetic eye disease group (node-splitting method).**

| **Side** |  | **Direct** |  | **Indirect** |  | **Difference** |  |  |  |
| --- | --- | --- | --- | --- | --- | --- | --- | --- | --- |
|  |  | **Coef.** | **Std.Err.** | **Coef.** | **Std.Err.** | **Coef.** | **Std.Err.** | **P>\|z\|** | **tau** |
| A | B | .1337392 | 4.866255 | 7.38948 | 6.069719 | -7.255741 | 7.779608 | 0.351 | 4.855966 |
| A | C | 5.343266 | .9957163 | 2.913208 | 6.464051 | 2.430058 | 6.540226 | 0.710 | 4.89423 |
| A | D | .5271802 | 1.556898 | 8.823866 | 3.201508 | -8.296685 | 3.560071 | **0.020** | 4.658906 |
| A | E | .5353978 | 1.590891 | 6.927869 | 3.172954 | -6.392471 | 3.55006 | 0.072 | 4.763995 |
| A | F | .2590564 | 1.699742 | 5.96733 | 3.25566 | -5.708273 | 3.672918 | 0.120 | 4.797868 |
| A | G | .7706915 | 2.411701 | 6.899681 | 3.809046 | -6.12899 | 4.508351 | 0.174 | 4.812928 |
| A | H | 4.717775 | 3.453146 | 9.921884 | 4.587622 | -5.204109 | 5.736805 | 0.364 | 4.860900 |
| A | I | .1299804 | 4.909282 | 2.514281 | 6.204351 | -2.3843 | 7.916933 | 0.763 | 4.896343 |
| A | J | .5845466 | 1.799282 | 7.237518 | 3.187009 | -6.652971 | 3.66002 | 0.069 | 4.752696 |
| A | K | .5933246 | 4.866305 | 7.849066 | 6.069642 | -7.255741 | 7.779608 | 0.351 | 4.855966 |
| A | L | 1.099933 | 4.866434 | 8.355674 | 6.069615 | -7.255741 | 7.779608 | 0.351 | 4.855966 |
| A | M | 2.546298 | 4.86723 | 9.802039 | 6.069880 | -7.255741 | 7.779608 | 0.351 | 4.855966 |
| A | N | .7664447 | 4.86634 | 8.022186 | 6.069625 | -7.255741 | 7.779608 | 0.351 | 4.855966 |
| A | O | .8849205 | 4.86637 | 8.140662 | 6.069619 | -7.255741 | 7.779608 | 0.351 | 4.855966 |
| A | P | 2.730737 | 2.791629 | 8.907281 | 3.943798 | -6.176543 | 4.829497 | 0.201 | 4.821878 |
| A | Q | .6841082 | 4.866322 | 7.939849 | 6.069632 | -7.255741 | 7.779608 | 0.351 | 4.855966 |
| A | R | .2763142 | 4.866263 | 7.532055 | 6.06969 | -7.255741 | 7.779608 | 0.351 | 4.855966 |
| A | S | .7618576 | 4.866339 | 8.017599 | 6.069626 | -7.255741 | 7.779608 | 0.351 | 4.855966 |
| A | T | .2947864 | 4.866265 | 7.550528 | 6.069686 | -7.255741 | 7.779608 | 0.351 | 4.855966 |

| A | U | 2.661759 | 4.867321 | 9.91750 | 6.069923 | -7.255741 | 7.779608 | 0.351 | 4.855966 |
| --- | --- | --- | --- | --- | --- | --- | --- | --- | --- |
| B | C | .3122112 | 4.890027 | 5.454239 | 6.13492 | -5.142028 | 7.84534 | 0.512 | 4.879777 |
| B | D | .4467177 | 4.899497 | -3.071975 | 6.445323 | 3.518693 | 8.096116 | 0.664 | 4.889252 |
| B | G | .0759658 | 4.908169 | -1.599702 | 7.308144 | 1.675668 | 8.803349 | 0.849 | 4.897970 |
| B | H | 1.649331 | 4.864776 | 11.21239 | 9.531728 | -9.563062 | 10.70169 | 0.372 | 4.854075 |
| B | J | .1117435 | 4.904873 | -2.369702 | 6.606024 | 2.481446 | 8.227828 | 0.763 | 4.894666 |
| B | P | .2640344 | 4.889388 | 5.799060 | 7.811233 | -5.535026 | 9.215272 | 0.548 | 4.879140 |
| C | D | .1326876 | 1.661661 | -9.644538 | 2.366129 | 9.777225 | 2.891017 | **0.001** | 4.386242 |
| C | E | -1.961734 | 1.569972 | -10.01951 | 3.323609 | 8.05778 | 3.676638 | **0.028** | 4.699700 |
| C | F | -2.182482 | 1.811561 | -7.816946 | 2.875440 | 5.634464 | 3.398949 | 0.097 | 4.781963 |
| C | G | -.1144361 | 2.778215 | -6.026564 | 3.101224 | 5.912128 | 4.163673 | 0.156 | 4.803439 |
| C | H | 4.457293 | 3.395241 | -4.344625 | 4.563533 | 8.801918 | 5.681095 | 0.121 | 4.781058 |
| C | I | .0423183 | 4.802524 | -11.12851 | 6.103279 | 11.17083 | 7.766288 | 0.150 | 4.793461 |
| C | J | -1.926963 | 1.829922 | -6.999574 | 3.386058 | 5.072611 | 3.849552 | 0.188 | 4.832184 |
| C | K | .147456 | 4.890016 | -4.994572 | 6.134915 | 5.142028 | 7.84534 | 0.512 | 4.879777 |
| C | L | .6541541 | 4.890077 | -4.487874 | 6.134969 | 5.142028 | 7.84534 | 0.512 | 4.879777 |

A, CCL1; B, CCL2; C, CCL3; D, CCL4; E, CCL5; F, CCL7; G, CCL8; H, CCL9; I, CCL11; J, CCL13; K, CCL15; L, CCL19; M, CCL20; N, CCL21; O, CCL22; P, CCL23; Q, CCL24; R, CCL25; S, CCL26; T, CCL27

# Table S6: Local inconsistency for the network of CXC chemokines in diabetic eye disease group (node-splitting method).

| **Side** |  | **Direct** |  | **Indirect** |  | **Difference** |  |  |  |
| --- | --- | --- | --- | --- | --- | --- | --- | --- | --- |
|  |  | **Coef.** | **Std.Err.** | **Coef.** | **Std.Err.** | **Coef.** | **Std.Err.** | **P>\|z\|** | **tau** |
| A | B | -.2637201 | 4.547972 | 2.397908 | 7.445716 | -2.661628 | 8.724662 | 0.760 | 9.091898 |
| A | C | .0680814 | 9.081116 | 4.190154 | 11.22775 | -4.122073 | 14.44055 | 0.775 | 9.075608 |
| A | D | -11.67823 | 7.721462 | 17.06125 | 10.45756 | -28.73948 | 13.04159 | **0.028** | 7.667468 |
| A | E | .2965728 | 6.404485 | 5.831986 | 9.502051 | -5.535414 | 11.459 | 0.629 | 9.052282 |
| A | F | 1.070623 | 9.081244 | 5.192696 | 11.22766 | -4.122073 | 14.44055 | 0.775 | 9.075608 |
| A | G | 4.644975 | 1.826886 | 11.26442 | 15.72131 | -6.619442 | 15.8268 | 0.676 | 9.014174 |
| A | H | -.5604009 | 3.958254 | 8.085301 | 6.491832 | -8.645702 | 7.607846 | 0.256 | 8.83854 |
| A | I | 3.003057 | 2.302595 | 13.84848 | 6.738455 | -10.84543 | 7.121764 | 0.128 | 8.906279 |
| A | J | .2167157 | 9.08112 | 4.338788 | 11.22773 | -4.122073 | 14.44055 | 0.775 | 9.075608 |
| A | K | .4410853 | 6.423618 | -.351861 | 9.284254 | .7929463 | 11.29027 | 0.944 | 9.080782 |
| A | L | .202268 | 9.08112 | 4.32434 | 11.22773 | -4.122072 | 14.44055 | 0.775 | 9.075608 |
| A | M | .1144652 | 9.081117 | 4.236538 | 11.22775 | -4.122072 | 14.44055 | 0.775 | 9.075608 |
| A | N | .971928 | 6.410942 | 5.690747 | 8.800073 | -4.718819 | 10.88754 | 0.665 | 9.060408 |
| B | C | -.0790023 | 9.072057 | 4.147138 | 13.47995 | -4.22614 | 16.24841 | 0.795 | 9.066543 |
| B | E | -.0820468 | 9.074087 | 2.977814 | 8.3704 | -3.059861 | 12.34514 | 0.804 | 9.068574 |
| B | F | .9235633 | 9.072152 | 5.149704 | 13.47992 | -4.22614 | 16.24841 | 0.795 | 9.066543 |
| B | G | .344888 | 5.068769 | 9.194561 | 5.733056 | -8.849673 | 7.65254 | 0.248 | 8.774527 |
| B | H | 1.178168 | 9.092905 | 1.441323 | 5.685528 | -.2631553 | 10.72409 | 0.980 | 9.087249 |
| B | I | 4.63805 | 4.520924 | 1.031897 | 7.91418 | 3.606154 | 9.116018 | 0.692 | 9.032509 |

| B | J | .0696356 | 9.072057 | 4.295775 | 13.47993 | -4.22614 | 16.24841 | 0.795 | 9.066543 |
| --- | --- | --- | --- | --- | --- | --- | --- | --- | --- |
| B | K | 1.350638 | 6.353798 | -7.153777 | 13.06275 | 8.504415 | 14.52692 | 0.558 | 8.982008 |
| B | L | .0551874 | 9.072057 | 4.281327 | 13.47993 | -4.22614 | 16.24841 | 0.795 | 9.066543 |
| B | M | -.0326175 | 9.072056 | 4.193523 | 13.47994 | -4.22614 | 16.24841 | 0.795 | 9.066543 |
| B | N | .6462842 | 6.366631 | 8.464917 | 13.04072 | -7.818632 | 14.51171 | 0.590 | 8.997943 |
| C | E | -.0030434 | 9.090709 | 1.814523 | 19.09803 | -1.817567 | 21.15126 | 0.932 | 9.085208 |

A, CXCL1; B, CXCL2; C, CXCL4; D, CXCL5; E, CXCL6; F, CXCL8; G, CXCL9; H, CXCL10; I, CXCL11; J, CXCL12; K, CXCL13; L, CXCL16; M, CX3CL1

# Figure S2. Forest plot of different chemokines between diabetic eye disease group patients and controls group.


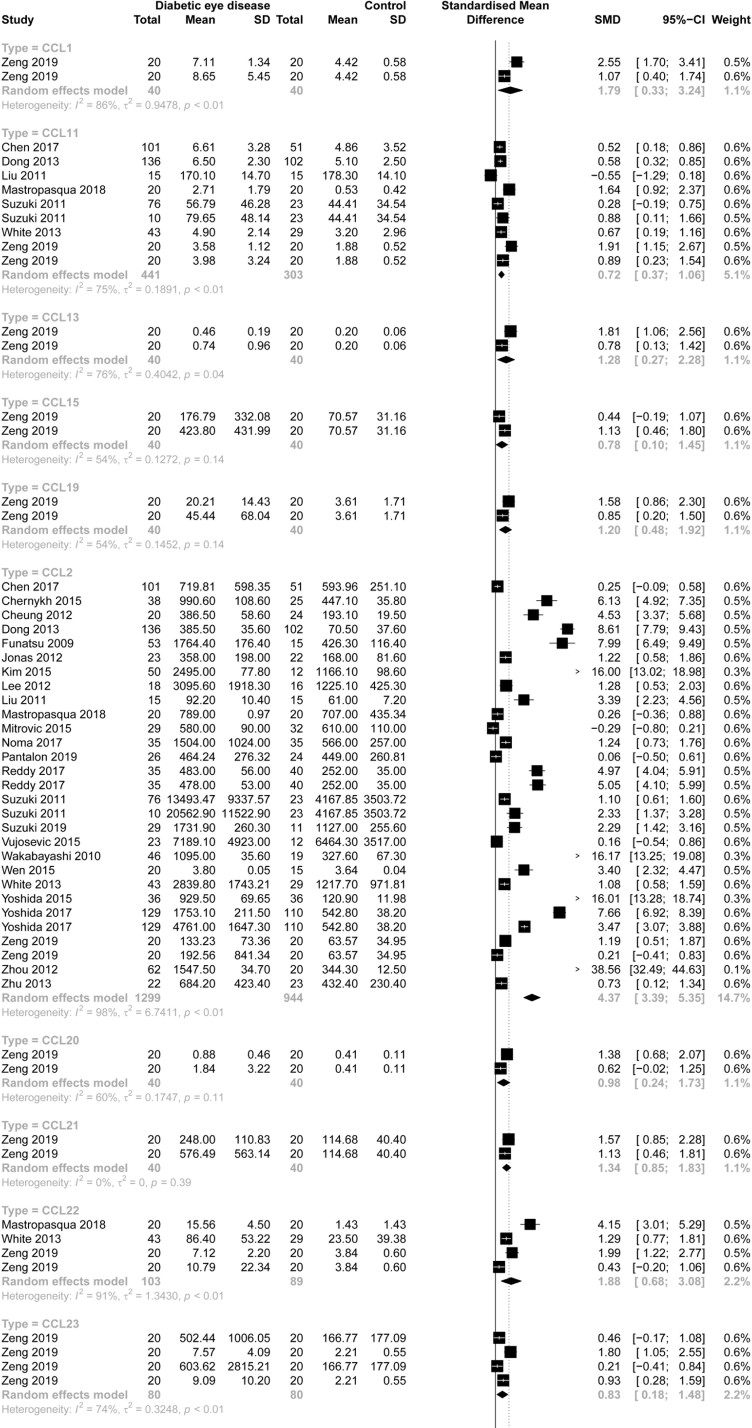


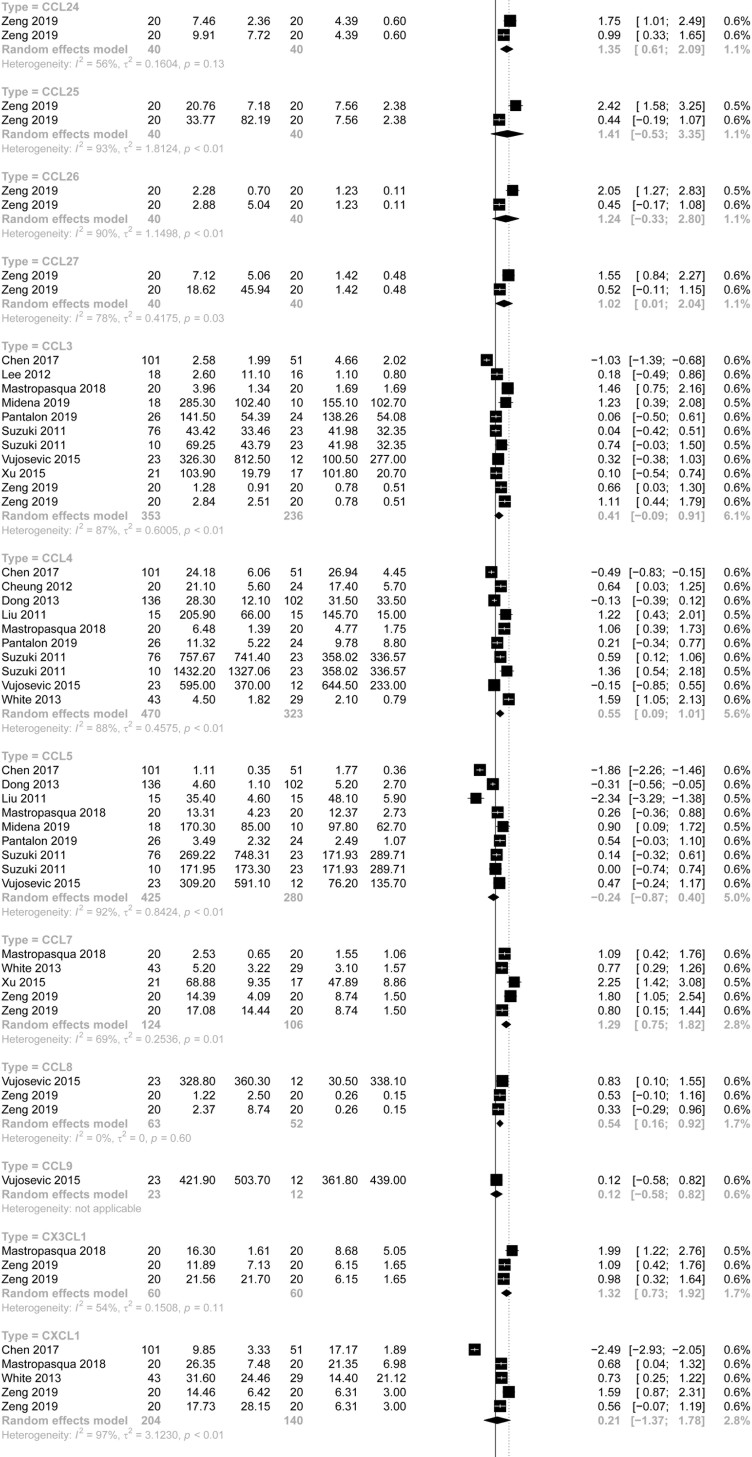


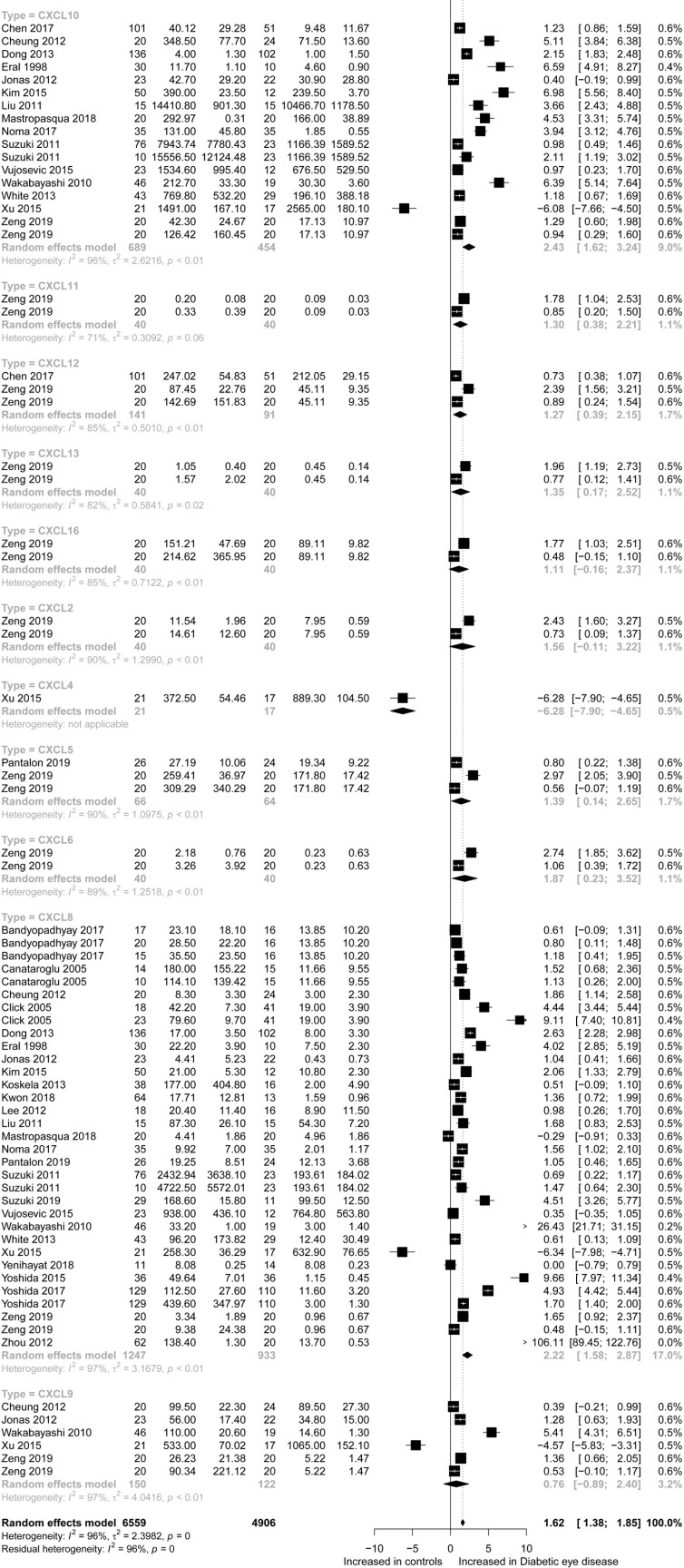


Study effect sizes of chemokines differences between diabetic eye disease group and controls. Each data marker represents a study, and the size of the data marker is proportional to the total number of individuals in that study. The summary effect size for each chemokines is denoted by a diamond. SMD, standardized mean difference.

# Table S7: Rank and SUCRA of the effect of different CC chemokines in diabetic eye disease.

| **Chemokines** | **SUCRA** | **MeanRank** |
| --- | --- | --- |
| **CCL8** | **81.3** | **4.7** |
| **CCL2** | **73.4** | **6.3** |
| CCL19 | 69.0 | 7.2 |
| CCL27 | 68.4 | 7.3 |
| CCL22 | 65.9 | 7.8 |
| CCL15 | 56.6 | 9.7 |
| CCL21 | 54.0 | 10.2 |
| CCL25 | 53.6 | 10.3 |
| CCL13 | 51.7 | 10.7 |
| CCL23 | 51.7 | 10.7 |

This table shows the relative cumulative probabilities for each of the chemokines in the network. SUCRA values are presented in the legend. SUCRA, surface under cumulative ranking curve.

# Table S8: Rank and SUCRA of the effect of different CXC chemokines in diabetic eye disease.

| **Chemokines** | **SUCRA** | **MeanRank** |
| --- | --- | --- |
| **CXCL8** | **73.3** | **4.5** |
| **CXCL10** | **69.0** | **5.0** |
| CXCL6 | 54.7 | 6.9 |
| CX3CL1 | 54.8 | 6.9 |
| CXCL5 | 51.0 | 7.4 |
| CXCL11 | 50.1 | 7.5 |
| CXCL13 | 50.1 | 7.5 |
| CXCL16 | 49.9 | 7.5 |
| CXCL2 | 49.5 | 7.6 |
| CXCL9 | 49.3 | 7.6 |

This table shows the relative cumulative probabilities for each of the chemokines in the network. SUCRA values are presented in the legend. SUCRA, surface under cumulative ranking curve.

# Figure S3: Egger funnel plots of diabetic eye disease patients compared to controls.


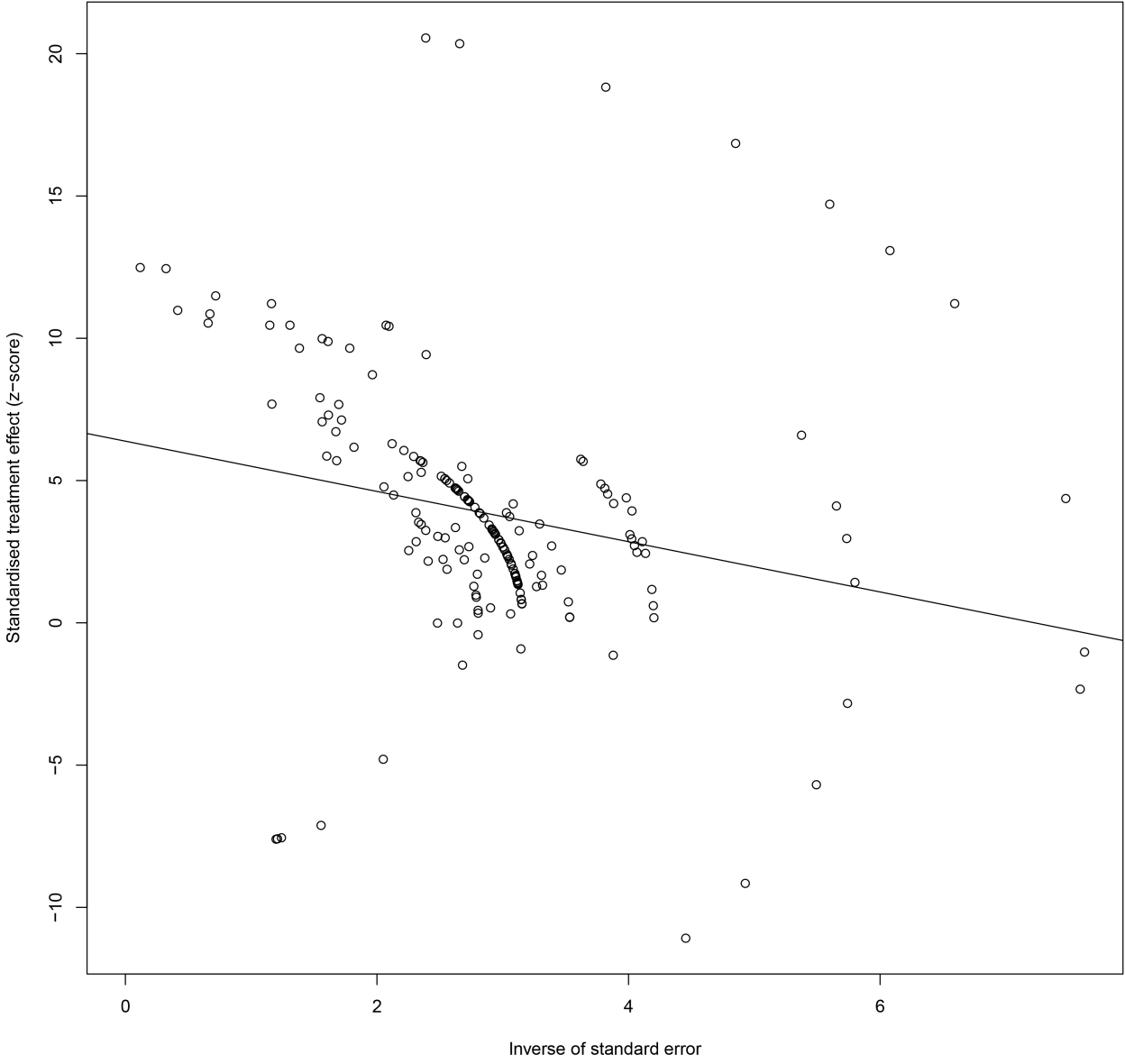


Egger funnel plots to assess publication bias (t = 1.326, p-value = 0.1178). Plots show study size as a function of effect size for studies included in the meta-analysis. The dots represent each study.

# Appendices References

1. Bandyopadhyay S, Bandyopadhyay SK, Saha M, Sinha A. Study of aqueous cytokines in patients with different patterns of diabetic macular edema based on optical coherence tomography. International ophthalmology. 2018; 38(1):241-249.

2. Canataroglu H, Varinli I, Ozcan AA, Canataroglu A, Doran F, Varinli S. Interleukin (IL)-6, interleukin (IL)-8 levels and cellular composition of the vitreous humor in proliferative diabetic retinopathy, proliferative vitreoretinopathy, and traumatic proliferative vitreoretinopathy. Ocular immunology and inflammation. 2005; 13(5):375-381.

3. Chen H, Zhang X, Liao N, Wen F. Assessment of biomarkers using multiplex assays in aqueous humor of patients with diabetic retinopathy. BMC ophthalmology. 2017; 17(1):176.

4. Chernykh VV, Varvarinsky EV, Smirnov EV, Chernykh DV, Trunov AN. Proliferative and inflammatory factors in the vitreous of patients with proliferative diabetic retinopathy. Indian journal of ophthalmology. 2015; 63(1):33-36.

5. Cheung CMG, Vania M, Ang M, Chee SP, Li J. Comparison of aqueous humor cytokine and chemokine levels in diabetic patients with and without retinopathy. Molecular Vision. 2012; 18(87-88):830-837.

6. Cicik E, Tekin H, Akar S, Ekmekçi OB, Donma O, Koldaş L*, et al*. Interleukin-8, nitric oxide and glutathione status in proliferative vitreoretinopathy and proliferative diabetic retinopathy. Ophthalmic research. 2003; 35(5):251-255.

7. Dong N, Xu B, Wang B, Chu L. Study of 27 aqueous humor cytokines in patients with type 2 diabetes with or without retinopathy. Mol Vis. 2013; 19:1734-1746.

8. Elner SG, Strieter R, Bian ZM, Kunkel S, Mokhtarzaden L, Johnson M*, et al*. Interferon-induced protein 10 and interleukin 8. C-X-C chemokines present in proliferative diabetic retinopathy. Archives of ophthalmology (Chicago, Ill : 1960). 1998; 116(12):1597-1601.

9. Funatsu H, Noma H, Mimura T, Eguchi S, Hori S. Association of vitreous inflammatory factors with diabetic macular edema. Ophthalmology. 2009; 116(1):73-79.

10. Bromberg-White JL, Glazer L, Downer R, Furge K, Boguslawski E, Duesbery NS. Identification of VEGF-independent cytokines in proliferative diabetic retinopathy vitreous. Invest Ophthalmol Vis Sci. 2013; 54(10):6472-6480.

11. Jonas JB, Jonas RA, Neumaier M, Findeisen P. Cytokine concentration in aqueous humor of eyes with diabetic macular edema. Retina (Philadelphia, Pa). 2012; 32(10):2150-2157.

12. Kim M, Kim Y, Lee SJ. Comparison of aqueous concentrations of angiogenic and inﬂammatory cytokines based on optical coherence tomography patterns of diabetic macular edema. Indian journal of ophthalmology. 2015; 63(4):312-317.

13. Koskela UE, Kuusisto SM, Nissinen AE, Savolainen MJ, Liinamaa MJ. High vitreous concentration of IL-6 and IL-8, but not of adhesion molecules in relation to plasma concentrations in proliferative diabetic retinopathy. Ophthalmic research. 2013; 49(2):108-114.

14. Kwon JW, Jee D. Aqueous humor cytokine levels in patients with diabetic macular edema refractory to anti-VEGF treatment. PLoS One. 2018; 13(9):e0203408.

15. Lee WJ, Kang MH, Seong M, Cho HY. Comparison of aqueous concentrations of angiogenic and inflammatory cytokines in diabetic macular oedema and macular oedema due to branch retinal vein occlusion. Br J Ophthalmol. 2012; 96(11):1426-1430.

16. Liu J, Shi B, He S, Yao X, Willcox MD, Zhao Z. Changes to tear cytokines of type 2 diabetic patients with or without retinopathy. Mol Vis. 2010; 16:2931-2938.

17. Mastropasqua R, D'Aloisio R, Di Nicola M, Di Martino G, Lamolinara A, Di Antonio L*, et al*. Relationship between aqueous humor cytokine level changes and retinal vascular changes after intravitreal aflibercept for diabetic macular edema. Scientific reports. 2018; 8(1):16548.

18. Midena E, Micera A, Frizziero L, Pilotto E, Esposito G, Bini S. Sub-threshold micropulse laser treatment reduces inflammatory biomarkers in aqueous humour of diabetic patients with macular edema. Scientific reports. 2019; 9(1):10034.

19. Mitrović S, Kelava T, Šućur A, Grčević D. Levels of Selected Aqueous Humor Mediators (IL-10, IL-17, CCL2, VEGF, FasL) in Diabetic Cataract. Ocular immunology and inflammation. 2016; 24(2):159-166.

20. Noma H, Mimura T, Yasuda K, Motohashi R, Kotake O, Shimura M. Aqueous Humor Levels of Soluble Vascular Endothelial Growth Factor Receptor and Inflammatory Factors in Diabetic Macular Edema. Ophthalmologica Journal international d'ophtalmologie International journal of ophthalmology Zeitschrift fur Augenheilkunde. 2017; 238(1-2):81-88.

21. Pantalon A, Obada O, Constantinescu D, Feraru C, Chiselita D. Inflammatory model in patients with primary open angle glaucoma and diabetes. Int J Ophthalmol. 2019; 12(5):795-801.

22. Reddy S, Amutha A, Rajalakshmi R, Bhaskaran R, Monickaraj F, Rangasamy S*, et al*. Association of increased levels of MCP-1 and cathepsin-D in young onset type 2 diabetes patients (T2DM-Y) with severity of diabetic retinopathy. Journal of diabetes and its complications. 2017; 31(5):804-809.

23. Suzuki Y, Nakazawa M, Suzuki K, Yamazaki H, Miyagawa Y. Expression profiles of cytokines and chemokines in vitreous fluid in diabetic retinopathy and central retinal vein occlusion. Japanese journal of ophthalmology. 2011; 55(3):256-263.

24. Suzuki Y, Yao T, Okumura K, Seko Y, Kitano S. Elevation of the vitreous body concentrations of oxidative stress-responsive apoptosis-inducing protein (ORAIP) in proliferative diabetic retinopathy. Graefe's archive for clinical and experimental ophthalmology = Albrecht von Graefes Archiv fur klinische und experimentelle Ophthalmologie. 2019; 257(7):1519-1525.

25. Vujosevic S, Micera A, Bini S, Berton M, Esposito G, Midena E. Proteome analysis of retinal glia cells-related inflammatory cytokines in the aqueous humour of diabetic patients. Acta Ophthalmol. 2016; 94(1):56-64.

26. Wakabayashi Y, Usui Y, Okunuki Y, Kezuka T, Takeuchi M, Goto H*, et al*. CORRELATION OF VASCULAR ENDOTHELIAL GROWTH FACTOR WITH CHEMOKINES IN THE VITREOUS IN DIABETIC RETINOPATHY. Retina-the Journal of Retinal and Vitreous Diseases. 2010; 30(2):339-344.

27. Wen J, Jiang Y, Zheng X, Zhou Y. Six-month changes in cytokine levels after intravitreal bevacizumab injection for diabetic macular oedema and macular oedema due to central retinal vein occlusion. Br J Ophthalmol. 2015; 99(10):1334-1340.

28. Xu Y, Cheng Q, Yang B, Yu S, Xu F, Lu L*, et al*. Increased sCD200 Levels in Vitreous of Patients With Proliferative Diabetic Retinopathy and Its Correlation With VEGF and Proinflammatory Cytokines. Invest Ophthalmol Vis Sci. 2015; 56(11):6565-6572.

29. Yenihayat F, Özkan B, Kasap M, Karabaş VL, Güzel N, Akpınar G*, et al*. Vitreous IL-8 and VEGF levels in diabetic macular edema with or without subretinal fluid. International ophthalmology. 2019; 39(4):821-828.

30. Yoshida S, Kubo Y, Kobayashi Y, Zhou Y, Nakama T, Yamaguchi M*, et al*. Increased vitreous concentrations of MCP-1 and IL-6 after vitrectomy in patients with proliferative diabetic retinopathy: possible association with postoperative macular oedema. British Journal of Ophthalmology. 2015; 99(7):960-966.

31. Yoshida S, Kobayashi Y, Nakao S, Sassa Y, Hisatomi T, Ikeda Y*, et al*. Differential association of elevated inflammatory cytokines with postoperative fibrous proliferation and neovascularization after unsuccessful vitrectomy in eyes with proliferative diabetic retinopathy. Clinical ophthalmology (Auckland, NZ). 2017; 11:1697-1705.

32. Zeng Y, Cao D, Yu H, Hu Y, He M, Yang D*, et al*. Comprehensive analysis of vitreous humor chemokines in type 2 diabetic patients with and without diabetic retinopathy. Acta Diabetol. 2019; 56(7):797-805.

33. Zhou J, Wang S, Xia X. Role of intravitreal inflammatory cytokines and angiogenic factors in proliferative diabetic retinopathy. Curr Eye Res. 2012; 37(5):416-420.

34. Zhu D, Zhu H, Wang C, Yang D. Intraocular soluble intracellular adhesion molecule-1 correlates with subretinal fluid height of diabetic macular edema. Indian journal of ophthalmology. 2014; 62(3):295-298.
